# Physicochemical Properties of Mechanochemical Activated HZSM‑5 Zeolite for Thermocatalytic Pyrolysis of Polypropylene

**DOI:** 10.1021/acsomega.5c07513

**Published:** 2025-12-26

**Authors:** Pedro F. A.C. Queiroz, Marcio D. S. Araujo, Edjane F. B. Silva, Aruzza M. M. Araujo, Amanda D. Gondim, Valter J. Fernandes, Antonio S. Araujo

**Affiliations:** † Program in Chemistry, Institute of Chemistry, 28123 Federal University of Rio Grande do Norte, Natal, RN 59078-970, Brazil; ‡ Laboratory of Environmental Analysis, Primary Processing and Biofuels, Institute of Chemistry, Federal University of Rio Grande do Norte, Natal, RN 59078-970, Brazil; § Laboratory of Catalysis and Petrochemistry, Institute of Chemistry, Federal University of Rio Grande do Norte, Natal, RN 59078-970, Brazil

## Abstract

In this work, we explore a mechanochemical approach using ball milling to synthesize hierarchical HZSM-5 zeolites, eliminating the need for hydrothermal processing. The synthesis was conducted using dual organic templates, cetyltrimethylammonium bromide (CTMABr), and tetrapropylammonium bromide (TPABr), under varying conditions of milling time, rotational speed, and solvent presence. The aim was to investigate the structural evolution of ZSM-5 under mechanical activation while avoiding amorphization. Characterization techniques including X-ray diffraction (XRD), Fourier transform infrared spectroscopy (FTIR), scanning electron microscopy (SEM), and nitrogen physisorption revealed that even five minute of milling induced significant modifications in the chemical structure and morphology. These changes involved recrystallization processes and the formation of new Si–O and – Al–O– bonds, resulting in restructured pore environments that may influence catalytic behavior. The best HZSM-5 sample mechanochemically activated was evaluated in the thermocatalytic pyrolysis of polypropylene at 475 °C in a fixed-bed reactor, under nitrogen as a gas carrier. After 30 min of reaction, the products were collected and analyzed by coupled gas chromatography and mass spectrometry. The catalyst demonstrated high selectivity toward monoaromatic hydrocarbons in the C_7_–C_9_ range, particularly alkylbenzene, toluene, xylene, and ethylbenzene. These results suggest a synergistic effect between the enhanced porosity and retained acidity of the modified zeolite, offering a promising route for the valorization of plastic waste into valuable hydrocarbons.

## Graphical abstract

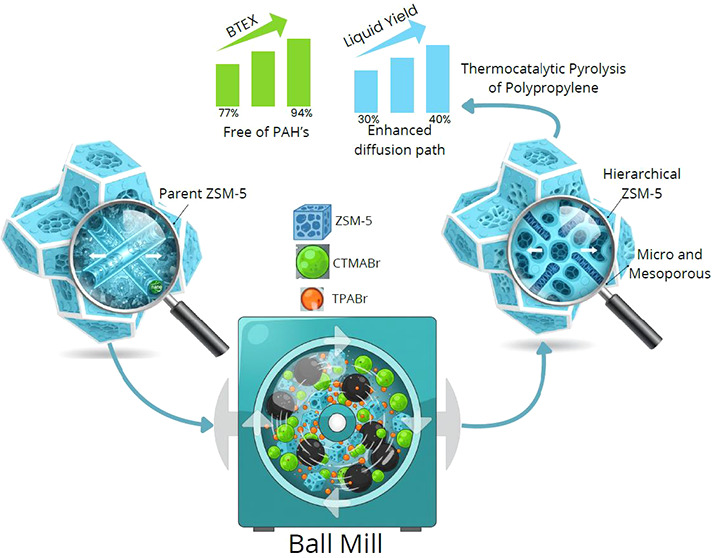

## 1 Introduction

ZSM-5 is recognized as a crystalline microporous material that has found applications in various processes. Its structure comprises a TO_4_ tetrahedron (T = Si or Al) within a three-dimensional (3D) system of micropores.^1^ Due to its shape-selective properties, ZSM-5 has been employed in the production of aromatics from biomass conversion through rapid catalytic pyrolysis. During this process, the larger molecules crack at the external site of the ZSM-5 bulky molecules and proceed to aromatization at the internal site. However, oxygenated molecules are too large to diffuse through the ZSM-5 small micropores, leading to pore blockage, increased coke formation, and eventual catalyst deactivation instead of producing more aromatics. One approach to address this issue is by introducing mesopores and macropores to enhance the catalyst’s surface area, allowing bulky molecules to diffuse across the ZSM-5 pore system.^2^

Hierarchical zeolites are crystalline porous materials defined by the presence of at least two distinct porosities: the intrinsic micropores (<2 nm) and a secondary porosity: mesopores (2–50 nm) or macropores (>50 nm).^3^ Crucially, to be recognized as hierarchical, the material must possess a highly interconnected network that provides continuous pathways linking the secondary pores to the micropores. This mandatory interconnection is what enables the catalyst to overcome diffusional limitations and is the key feature that distinguishes a hierarchical material from a simple mixture of different pore sizes.^4^

Secondary pore formation can be achieved during zeolite synthesis or postsynthesis, typically through hydrothermal methods involving either base treatment (desilication) or acid treatment (dealumination). These methods entail partial destruction of zeolite by removing silicon and aluminum atoms using solvents, heat, and often lengthy durations. This method can be replaced by generating energy through mechanical collisions directly on the catalyst surface, with ball milling being a prominent technique involving high-speed ball-material impacts. This technique minimizes or eliminates solvent usage and reduces the synthesis time of hierarchical zeolites.^5^

Mechanochemistry refers to the ability to accumulate and direct energy through mechanical forces, such as compression, grinding, and shearing, to facilitate reactions without solvent or heat addition. Mechanical forces can open new reaction pathways inaccessible through conventional methods.^6^ Mechanochemical reactions involve precursors being crushed between grinding balls, accumulating sufficient energy to break chemical bonds and generate surface defects. Mechanochemical synthesis yields new materials or structural modifications that are induced in the reactants during milling. Simplicity, cleanliness, cost-effectiveness, and high efficiency of mechanochemistry ensure its utility in synthesizing hierarchical materials, promoting the formation of meso-macropores.^7^

The goal of this work is illustrated in Figure 1. Optimize and develop hierarchical pores in ZSM-5 zeolite by treating it with two templates: tetrapropylammonium bromide (TPABr) and cetyltrimethylammonium bromide (CTMABr), in the absence of solvent, using mechanochemistry to introduce meso-macropores into ZSM-5 microporous structure for application in processing bulky molecules. These bulky molecules will be evaluated in the pyrolysis of polypropylene, and the improvements will be observed compared with the commercial ZSM-5.

**Figure 1:**
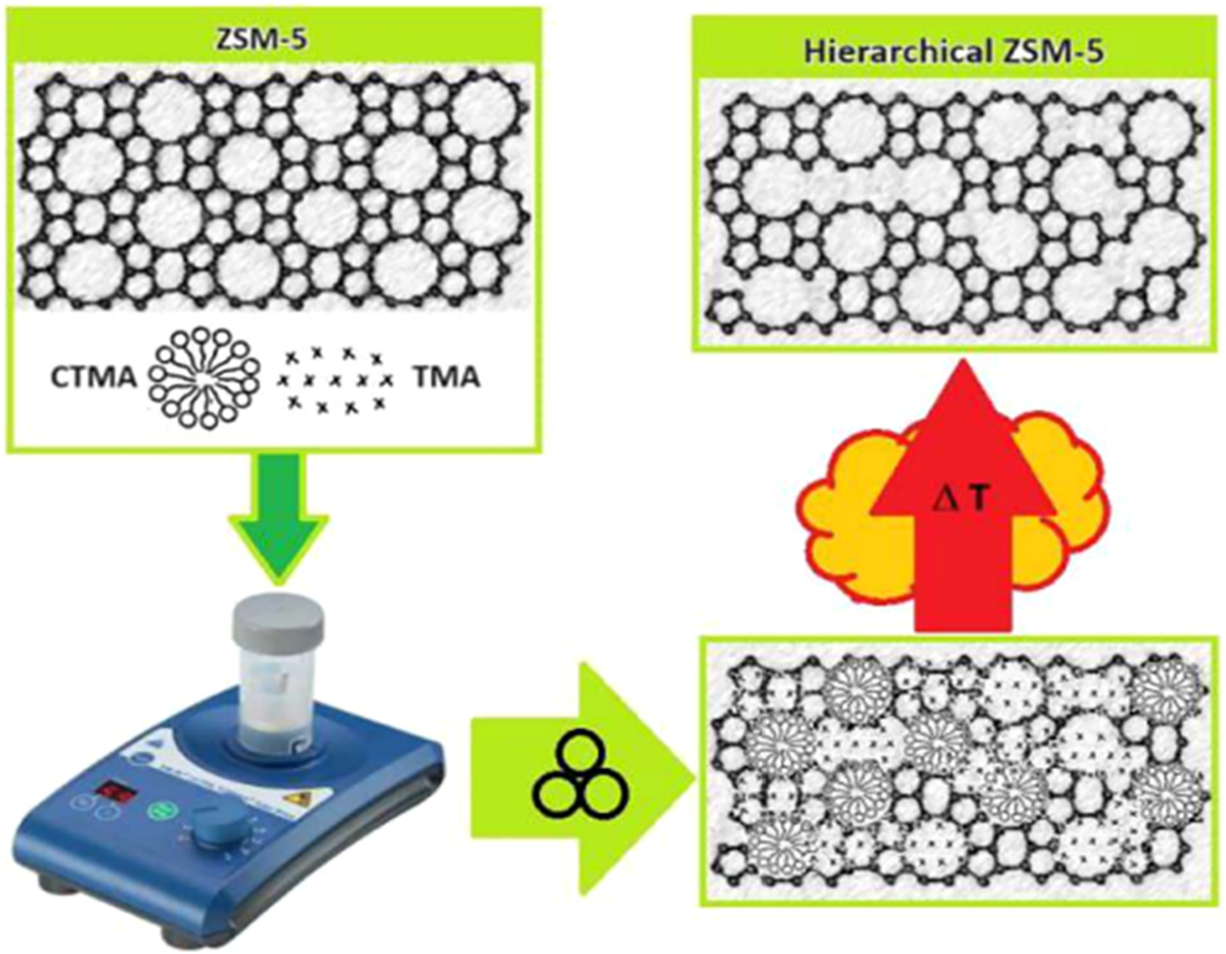
Proposed scheme using IKA reactor, for development of mesoporosity and secondary porosity in HZSM-5 zeolite utilizing cotemplates: CTMA^+^ and TPA^+^ by mechanochemistry activation and template removal during calcination (the IKA reactor Ultra Turrax Tube Drive (UTTD) is adapted with permission from IKA Werke GmbH & Co. KG. Copyright 2025 IKA Werke GmbH & Co. KG).

## 2 Experimental Section

### 2.1 Materials and Reagents

NH_4_ZSM-5 zeolite from *Zeolyst* with a Si/Al ratio of 23 was employed. Organic templates used were a solid powder of cetyltrimethylammonium bromide ≥98% (CTMABr) and a solid powder of tetrapropylammonium bromide 99% (TPABr), both supplied by Sigma-Aldrich. Deionized water and ethanol P.A. were obtained from Sigma-Aldrich. Polypropylene (PP) was provided by the Industry of Thermoplastic materials.

### 2.2 Mechanochemical Activation

Mechanochemical activation of the NH_4_ZSM–5 zeolite was performed using an Ultra Turrax Tube Drive (UTTD) IKA Instruments ball mill. This equipment, fitted with a mechanochemical reactor and 6 mm stainless steel balls, operates at speeds ranging from 300 to 6000 RPM, regulated by a rotation scale of 0 to 9.

#### 2.2.1 Experimental Optimization of the Ball Milling Procedure

Due to the scarcity of literature regarding the use of this specific equipment for zeolite mechanochemical modification, extensive optimization tests were required to determine the optimal milling parameters. All optimization experiments utilized 1 g of the NH_4_ZSM–5 zeolite in the presence of structure-directing agents (SDA): CTMABr and TPABr each loaded at 10 wt % relative to the zeolite mass. Specially, 0.10 g of CTMABr and 0.10 g of TPABr were used for every 1.0 g of NH_4_ZSM–5 zeolite within the reactor vessel under continuous milling. The parameters varied included the milling medium (aqueous, ethanol, solvent-free, and 5 drops of ethanol), the number of milling balls, and the rotation speed. The full set of initial experimental conditions is summarized in Table 1.

**Table 1:** Experimental Design for Determining the Optimal Conditions of the Ultra Turrax Tube Drive UTTD Mechanochemical Reactor; All Samples Underwent 20 Minutes of Milling

sample	grinding balls	rotation (rpm)	solution
ZSM-5–0	0	0	Pure^c^
ZSM-5-Y	^a^	3000	5 mL water
ZSM-5-Z	15	^b^	Pure^3^
ZSM-5–1	6	1000	10 wt % CTMA + TPA and 5 mL ethanol
ZSM-5–2	6	3000	10 wt % CTMA + TPA and 5 mL ethanol
ZSM-5–3	18	3000	10 wt % CTMA + TPA^c^
ZSM-5–4	15	3000	10 wt % CTMA + TPA^c^

a The number of grinding balls of stainless steel varied.

b varied multiple rotation scale.

c No solvent was used.

After milling, distilled water was added into the reactor vessel and kept under constant stirring for two min at low rotation scale, followed by filtration and drying at room temperature for 24 h. The samples were calcined at 550 °C, at a heating rate of 10 °C/min for 5 h, 1 h under N_2_ atmosphere 100 mL/min and 4 h in synthetic air. After calcination, the samples were stored and labeled as follows: ZSM-5-X, where *X* denotes the variables that were modified. The prefix “Hi-” was used in the study to indicate the activation milling time under optimal conditions found out previously. The commercial zeolite is designated as ZSM-5–0.

#### 2.2.2 Time-Dependent Study at Optimal Conditions

After the optimal conditions were found, we developed a time-dependent study, presented in Table 2, performed under the optimal conditions determined in the previous study, to specifically evaluate the effect of the ball milling duration on the development of hierarchical zeolites. The same proportion of zeolite and SDA was used.

**Table 2:** Mechanochemical Activation of Commercial ZSM-5 with 10 wt % CTAB and TPA as a Function of Milling time^a^

sample	time (min)	solution	grinding balls	RPM
Hi-ZSM-5–5	5	solid	15	3000
Hi-ZSM-5–10	10	Solid	15	3000
Hi-ZSM-5–15	15	solid	15	3000
Hi-ZSM-5–20	20	solid	15	3000

a All samples were treated in the best conditions optimized previously.

Subsequently, all samples were calcined at 550 °C, initially for 1 h under nitrogen gas presence and then for an additional 4 h in synthetic air. After calcination, the samples were stored and labeled as follows: ZSM-5-X, where *X* represents the milling time. The commercial zeolite is designated as ZSM-5–0. The prefix “Hi” was used in the study to indicate the milling time under the optimal milling conditions.

### 2.3 Physicochemical Characterizations

X-ray diffraction (XRD) analyses were conducted using a Bruker D2Phaser instrument equipped with a Lynexye detector and copper radiation (Cu Kα λ = 1.54Å) with a nickel filter, operating at 30 kV voltage and 10 mA current, utilizing a Lynxeye detector in the 2θ range within 3–50°. The XRD data were able to calculate the crystallite size through *Scherrer’s* equation, *d*_(hkl)_ (eq 1). The Relative Crystallinity RC (%) eq 2 was defined based on the intensity of the characteristic peaks (after background subtraction) in the 2θ of 22.5–25° of the ZSM-5 structure with mechanical activation, having the commercial ZSM-5 as 100% crystallinity. The Müller indices [(101), (200), (501), (151), and (133)] were used as the foundation for the primary diffractogram peaks, and the crystallinity indices were obtained from the mean of these intensities.

1 $$d_{\left(hkl\right)}=\frac{k\lambda }{\beta \;\cos\theta }$$

where *d*_(hkl)_ is the crystallite size (nm); ***k*** is the Scherrer’s constant (0,89); ***λ*** is the wavelength for Cu Kα radiation (λ = 0,1541 nm); **β** is known as Full Width Half Maximum (FWHM), measured in radians, and θ is Bragg’s diffraction angle. The FWHM was determined from the most intense peak of the zeolite according to the Müller indices (hkl).

2 $$\mathrm{RC}\left(\%\right)=\frac{\sum I_{\mathrm{sample}}}{\sum I_{\mathrm{standart}}}\times 100$$

where *I*_standart_ = total intensity of the peaks of the standard ZSM-5 sample, and *I*_sample_ = total intensity of the samples.

The scanning electron microscopy (SEM) images were obtained with a TESCAN MIRA 4 instrument using a secondary electron in-Beam SE detector with 10 KeV energy. Fourier Transform Infrared (FTIR) analyses were performed using a PerkinElmer Frontier model instrument with a resolution of 4 cm^–1^, covering the spectral absorption range of 400–4000 cm^–1^. Nitrogen adsorption–desorption was carried out at 77 K using a Micrometrics ASAP 2020 instrument. The surface area analysis was conducted using the Brunauer–Emmett–Teller (BET) method.

The total acidity was determined by n-butylamine adsorption, as a molecule probe, followed by desorption using thermogravimetry.^8^ The material chosen for acidity thermal desorption and catalyst test was Hi-ZSM-5–20, the sample treated with cotemplates during 20 min of ball milling. This sample showed better results and most promised to have better results on the desired properties.

In this analysis, approximately 0.1 g of each sample is first activated under a N_2_ flow (100 mL/min) for 2 h. Then, *n*-butylamine vapors are introduced to the sample at 95 °C for 60 min, ensuring full saturation of the catalyst’s acidic sites. The sample, now containing the amine, is purged with pure N_2_ at the same adsorption temperature for an additional hour to remove any physisorbed base. Desorption of n-butylamine begins by heating around 10 mg of the saturated sample in a METTLER TGA/SDTA 851 thermobalance at a rate of 10 °C/min up to 900 °C in a nitrogen atmosphere. Acidic sites were determined by the amount of desorbed *n*-butylamine, in millimoles, normalized by the sample mass, based on the thermogravimetric desorption curves of *n*-butylamine. The total stable acidic sites (*N*_sites_) chemisorbed by *n*-butylamine, in mmol/g, were used to calculate the Total Acidity (*A*_total_). The number of absorbed acids (mol/g) was calculated by using the mass of thermally desorbed *n*-butylamine (g) in each event, divided by the product of the molecular weight of the base (MM_*n*‑butylamine_ = 73.14 g/mol) and the mass of the catalyst free of *n*-butylamine (g), according to eq 3.^9^

3 $$A_{\mathrm{total}}=\sum N_{\mathrm{sites}}\frac{m_{n‐\mathrm{butylamine}}}{{\mathrm{MM}}_{n‐\mathrm{butylamine}}\times M_{\mathrm{catalyst}}}$$

### 2.4 Catalyst Test

The pyrolysis processes were carried out with 10 g of PP using N_2_ as a gas carrier flowing at a rate of 100 mL/min. For thermocatalytic pyrolysis, 10% by weight of the catalyst was used. The thermal and thermocatalytic pyrolysis of PP were carried out in the fixed-bed bench reactor (see Figure 2), model FT-1200, from the manufacturer Furnace INTI with a heating rate of 30 °C/min up to a temperature of 475 °C for 30 min in the thermal pyrolysis of PP, the aim was to observe the behavior of the pellet depending on the formation of pyrolysis oil and whether the low contact surface influences the degradation of PP. Thermocatalytic degradation of PP, using commercial and mechanochemistry modified ZSM-5 zeolite, was carried out under the same experimental conditions as for individual PP, to analyze the chemical interaction between the pellets and the catalyst.

**Figure 2:**
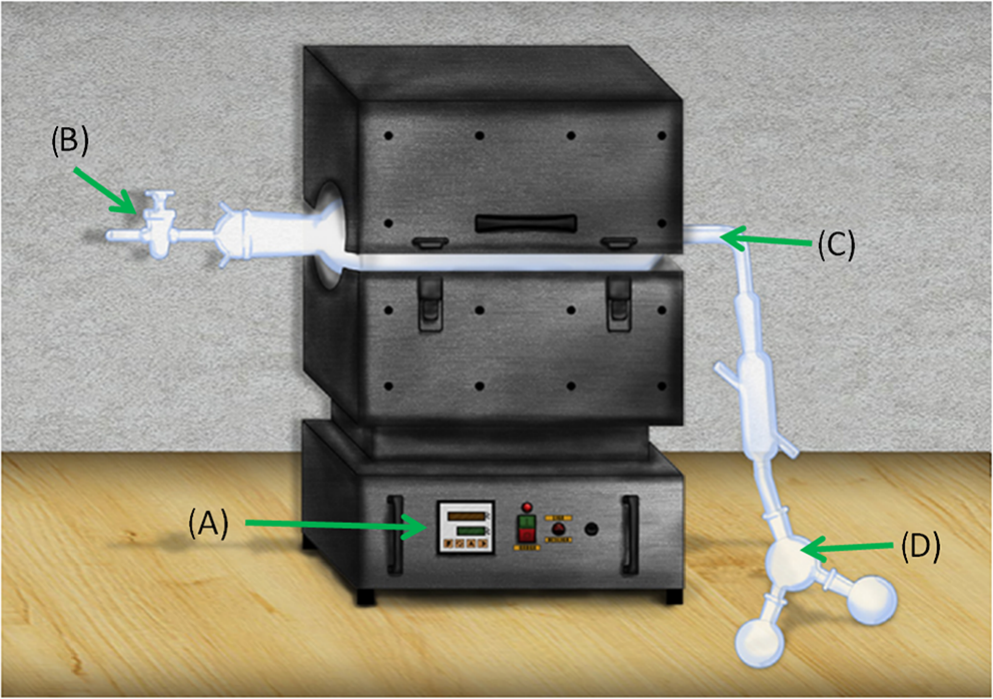
(A) FT-1200 fixed-bed reactor for pyrolysis. (B) Gas connection. (C) Pyrex connection. (D) Ice-cold bath. (Adapted with permission from Araújo, A. M. de M.; Lima, R. de O.; Gondim, A. D.; Diniz, J.; Souza, L. D.; Araujo, A. S. de. Thermal and Catalytic Pyrolysis of Sunflower Oil Using AlMCM-41. *Renew. Energy* **2017**, *101*, 900–906. Copyright 2017 Elsevier.)^10^

The vapors generated during degradation with and without catalysts were cooled through the connection of the flask (*C*) involving the pyrolysis reactor (*A*) and the flask in an ice-cold bath at 4 °C (*D*); consequently, the lighter fraction was deposited in the flask in an ice-cold bath while the heaviest fraction was retained in the flask connection. Then, the pyrolysis liquid was subjected to thermogravimetric analysis (TGA) to observe the thermal behavior and analyzed by gas chromatography-mass spectrometry (GC-MS).

The coke mass is determined by the difference in mass of the crucible before and after the pyrolysis process. The liquid yield is calculated by dividing its respective mass by the initial mass of the reactant, as shown in eq 4. The noncondensable gas weight is calculated in eq 5 by the difference in mass between the coke and the pyrolysis oil.

4 $$yield\;oil\;\left(\%\right)=\frac{condensed\;gasses}{initial\;mass\;of\;PP}\times 100$$

5 yieldgas(%)=100−[%coke+%yieldoil]

The pyrolysis oil samples were analyzed using gas chromatography–mass spectrometry (GC-MS) (Thermo Scientific ISQ) equipped with a DB-5MS column (30 m × 0.25 mm × 0.25 μm). Helium (1.0 mL/min) served as the carrier gas. A 1 μL sample was injected at 250 °C. The oven temperature program was 40 °C (4 min), ramped to 270 °C at 8 °C/min (5 min), and ramped to 300 °C at 10 °C/min (20 min). The mass spectrometer utilized EI mode (70 eV), with the source and transfer line maintained at 250 °C. Compound identification was achieved by comparing mass spectra against the NIST Library (Version 2.0). Quantification was performed by normalizing the area of each compound to the total chromatographic area.

## 3 Results and Discussion

### 3.1 Crystallographic Properties

Figure 3 depicts the X-ray diffraction (XRD) pattern of the mechanochemically activated commercial ZSM-5 zeolite, with variations in factors such as ball quantity, rotation scale, time, and the use of organic directing agents and solvent. All samples exhibit clear crystallinity and display intensive diffraction peaks, corresponding to peaks at 7–9 and 22–25° angles of the ZSM-5 zeolite structure under all reaction conditions, indicating highly crystallinity nature.^11^ Emphasis is placed on the ZSM-5-Y sample, which was solely treated under milling, with 5 mL of water without a directing agent, showing a slight decrease in crystallinity, indicating that milling affects the surface of ZSM-5 without significant losses in relative crystallinity, demonstrating that ball milling provides sufficient energy to affect the chemical and physical structure of ZSM-5.

**Figure 3:**
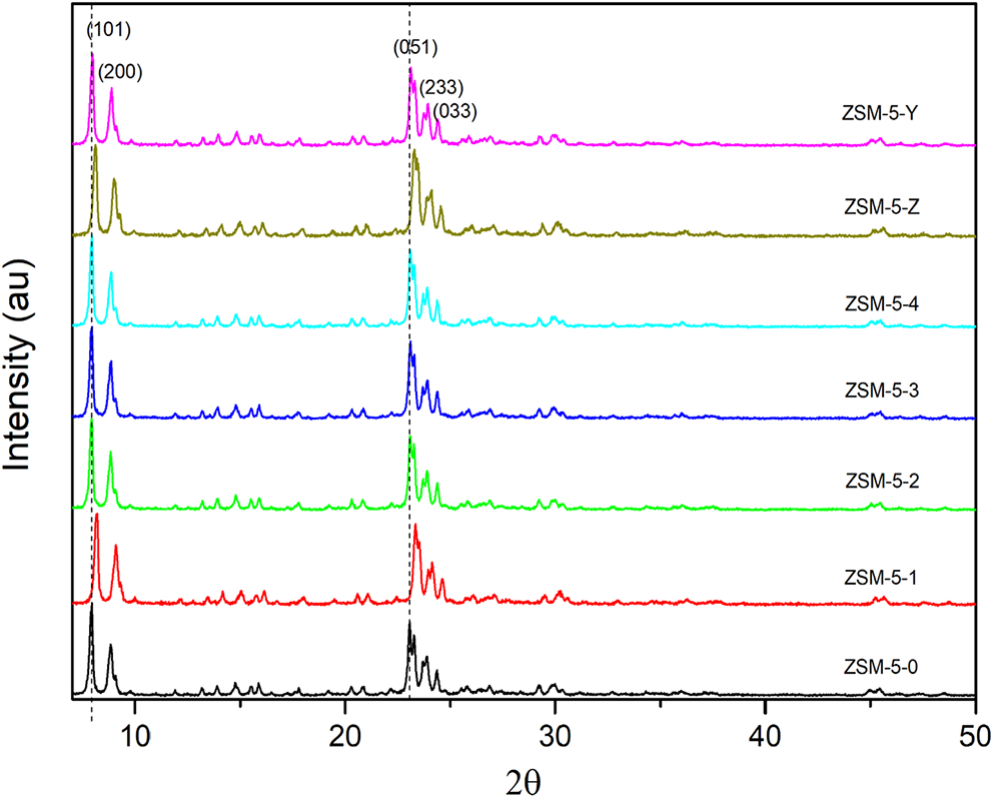
X-ray diffraction (XRD) patterns of zeolites under different mechanochemical treatments. ZSM-5–0 corresponds to untreated commercial zeolite without directing agents.

Crystallite size was calculated using the Scherrer equation.^12,13^ Variations in the crystallite size and a decrease in relative crystallinity suggest modifications to the ZSM-5 micropores and nanocrystals. The diffractogram of ZSM-5-Z confirms this reduction in the crystallite size. To identify the minimum required rotation speed and ball quantity for effective milling, the experimental rotation scale and ball quantity were systematically varied. It was determined that a minimum of six stainless steel balls are necessary to ensure collision with the sample at five rotation scales (3000 RPM). ZSM-5–1 was conducted in the presence of 5 mL of ethanol as a solvent, showing a decrease in crystallite size at a low rotation scale, indicating structural modification only with solvent and directing agent treatment, without energy supply from ball collisions, as in this rotation scale, it was not possible to overcome the inertia of the grinding balls to induce collisions between the samples and the walls of the reaction vessel.

The ZSM-5–2 activation was performed under the same conditions as ZSM-5–1, but at a higher rotation scale (5, equivalent to 3000 RPM) to ensure high-frequency collisions between the grinding balls and the sample. Despite the increased mechanical energy input, neither the crystallite size nor the relative crystallinity exhibited significant variation. This stability is likely due to an enhanced recrystallization effect facilitated by the presence of templates within the ethanolic medium during milling.

For the ZSM-5–3 experiment, a high rotation scale (3000 RPM) and an excessive number of grinding balls were used without solvent to maximize collisions among the balls, the sample, and the templates. Since no considerable change was observed in relative crystallinity (and only a minor 1.5% increase in crystallite size), we suggest that the excess grinding media primarily intensified ball-to-ball collisions. This reduced the effective collision frequency between the grinding balls and the sample, consequently increasing the amount of energy dissipated as heat (thermal energy) rather than mechanical force. Therefore, the conditions likely favored a temperature increase over inducing the desired template-zeolite reaction through direct mechanical forces.^14^

The ZSM-5–4 treatment conditions were identified as optimal based on the initial observed decrease in relative crystallinity. This drop signifies that the mechanical forces achieved the critical energy threshold to initiate the full, three-stage mechanochemical process: surface collision, elastic and plastic deformation followed by the desired final chemical reactions.^15^

The high grinding energy generates transient radicals and provides sufficient energy for TPA^+^ to overcome its weak basicity and cleave the surface siloxane (Si–O–Si) bonds. This critical scission creates structural defects and voids, allowing both TPA^+^ and CTMA^+^ to diffuse into the microcavities. There, templates form new chemical bonds deep within the zeolite structure. At longer durations, such as 20 min, the material enters a state of dynamic equilibrium. The continuous variation in crystallite size and the net reduction in relative crystallinity reflect the interplay between continuous bond breaking (destruction) and simultaneous recrystallization (reorganization).^16^ The crystallographic properties resulting from this dynamic process are detailed in Table 3.

**Table 3:** Crystal Size Calculated by Scherrer’s Equation and Relative Crystallinity

sample	crystallite size (nm)^a^	relative crystallinity (%)^b^
ZSM-5–0	40.8	100
ZSM-5-Y	41.4	90
ZSM-5-Z	38.5	100
ZSM-5–1	37.5	100
ZSM-5–2	41.3	100
ZSM-5–3	41.3	99
ZSM-5–4	40.9	92
Hi-ZSM-5–5	36.2	74
Hi-ZSM-5–10	40.8	83
Hi-ZSM-5–15	41.5	81
Hi-ZSM-5–20	40.1	76

a Crystallite size estimated by Scherer’s equation for the peaks between 2θ = 7–10°.

b Relative crystallinity calculated based on the sum of peak areas between 2θ = 22–25° from XRD pattern of hierarchical ZSM-5 samples compared IZA’s MFI: XPD pattern calcined HZSM-5 100% crystallinity.

In sample ZSM-5–4, a decrease in relative crystallinity is observed, showing that treatment these conditions were optimal because it is sufficient to break the Siloxane (Si–O) bond and create space for TPA^+^ and CTMA^+^ to diffuse and fill the space generated by milling. At 20 min, the variation in crystallite size occurs due to continuous recrystallization and breaking processes, causing a reduction in relative crystallinity and shifting in the catalyst peaks. The crystallographic properties of the modified materials are given in Table 3.

Figure 4 presents the XRD diffractograms as a function of milling time, and Table 3 indicates that structural modifications can occur in as little as 5 min of grinding. However, increasing the milling time under the optimal reactor conditions reveals a dynamic equilibrium within the material. The CTMA and TPA agents are key to this process: CTMA promotes subsequent recrystallization and structural reorganization, while TPA provides a protective effect that enhances overall crystal integrity. This interplay between the mechanical force (breaking crystalline structures) and the chemical agents (reorganizing structures) results in the observed nonlinear behavior of the relative crystallinity and crystallite size, which initially drop and then either stabilize or increase with prolonged grinding.^17^ Longer grinding could lead to contamination between the grinding balls and the zeolite.^18,19^

**Figure 4:**
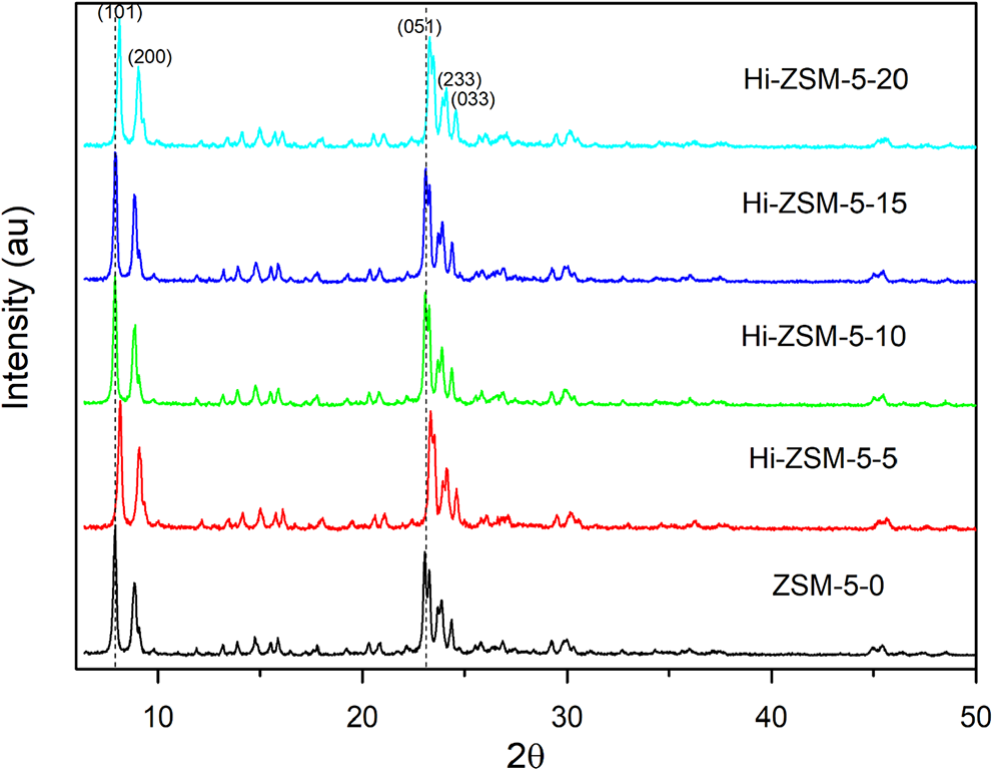
XRD patterns of the zeolites activated by mechanochemical treatment as a function of rotation time (0, 5, 10, 15, and 20 min). The ZSM-5–0 sample represents the commercial zeolite.

In terms of peak positions, all samples exhibited similar characteristics to ZSM-5, albeit slightly shifted toward higher angles compared to the commercial zeolite. The Hi-ZSM-5–10 and Hi-ZSM-5–15 samples showed an increase in crystallite size, calculated using the Scherrer equation, suggesting that after milling, TPABr protects and stabilizes the ZSM-5 silica, aiding in maintaining the crystalline structure.^20^ Howbeit, in the Hi-ZSM-5–5 and Hi-ZSM-5–20 samples, a decrease in crystallite size was observed, indicating that treatment for just 5 min is sufficient to induce cleavage in the siloxane (Si–O–Si) surface bonds and allow the TPA and CTMA to diffuse into the microcavities causing restructuring and recrystallization inside the pores. With milling times exceeding 5 min, recrystallization and breakage occur, leading to an increase in crystallite size with longer milling durations.

The right shift (lattice contraction) in the XRD pattern (to a higher 2θ angle) is primarily attributed to two concurrent phenomena induced by ball milling: the reduction in crystallite size and the subsequent crystal agglomeration. Mechanical activation increases the superficial tension, which in turn causes a contraction of the unit cell and a reduction in the interplanar spacing. Furthermore, the final removal of the organic templates during calcination may induce a secondary contraction of the crystalline network due to the evacuated microporosity volume. This overall process favors the development of nanocrystals with high surface energy, which can subsequently promote localized recrystallization.^21–23^

Conversely, a left shift (Lattice Expansion) in the XRD pattern (to a lower 2θ angle) indicates an expansion of the crystalline lattice, representing an increase in the interplanar spacing. This expansion is typically caused by the physical presence of bulky organic molecules or hydrocarbon fragments trapped within the microporous channels, which exerts an internal pressure on the framework. For instance, the samples ZSM-5–2 and Hi-ZSM-5–10 exhibit this shift, pointing to a significant accumulation of residual organics inside the micropores that cause the observed structural expansion.^24,25^

### 3.2 Structural Properties by Infrared Spectroscopy

Figure 5 displays the FTIR transmittance spectrum of commercial NH_4_ZSM-5 and the mechanochemically activated material. The absorption bands are situated between 1400 and 400 cm^–1^, with no significant bands observed before this range. The typical ZSM-5 bands that appear at 1225 cm^–1^ are found in well crystallized zeolite and are presented in all samples, related to asymmetric stretching T-O-T of the vibrational tetrahedral.^26^

**Figure 5:**
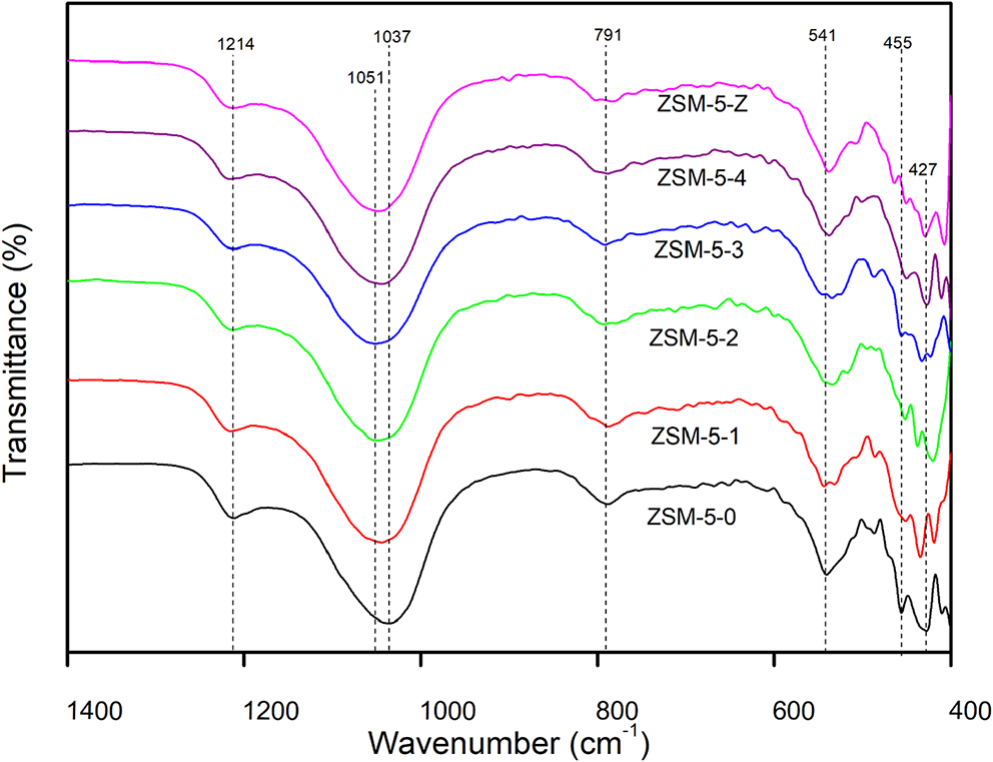
Fourier Transform Infrared (FTIR) spectra of zeolites under different mechanochemical treatments. The ZSM-5–0 corresponds to the commercial zeolite.

The strong band originally observed at 1035 cm^–1^ corresponds to the asymmetric Si-O-Si stretching vibrations within the internal framework T­(Al/Si)­O_4_ tetrahedra.^27^ Following the mechanochemical treatment, this primary framework band undergoes a slight shift to 1051 cm^–1^. This shift is principally attributed to the deformation in T–O–T angles caused by elastic tensions and stress induced by the grinding process. Moreover, changes in the Si/Al ratio within the structure can also influence the band’s position.^28^ Overall, such shifts in IR absorption bands are commonly associated with the loss in relative crystallinity induced by the mechanical grinding.^29^ The shift to a higher wave is due the presence of other cations, suggesting the varying in the Si/Al ratio once the bond distance of Si–O is shorter than Al–O.^30^ Figures S1 and S2 show energy-dispersive X-ray spectroscopy (EDS) elemental mapping and spectra and Table S1 summarized the Si/Al ratio calculated by EDS that confirms the increase of Al ratio related to the higher shift.

The band at 791 cm^–1^ corresponds to symmetric stretching of the internal and external tetrahedral bands of the T–O bond, respectively. Bands at 540 cm^–1^ represent asymmetric stretching of the five-membered ring (D5R),^31^ however sharp bands between 541 and 472 cm^–1^ are also related to bending or motion of the external linkage of AlO_4_ and SiO_4_ tetrahedra while medium intensity bands is from D-4 and D-6 rings, this frequency is assigned to a breathing motion of the isolated rings forming pore opening in zeolites.^32,33^ Also, these bands are sensitive to the crystallinity of the sample and a decrease in intensity is due the mechanical activation as supported in XRD data.^34^

Both XRD and FTIR were decisive in developing optimal conditions that were further applied to study the influence of time in grinding at optimal conditions. The optimal conditions are 15 stainless steel balls of 6 mm, without solvent and a scale of 5 (3000 RPM) in the UTTD ball mill reactor to induce ball milling. The results presented as a function of time were performed in the optimal condition.

Figure 6 presents the FTIR spectrum as a function of time. After 5 min (Hi-ZSM-5–5) of milling, a band at 900 cm^–1^ emerges, which becomes more pronounced after 10 min of milling, corresponding to the stretching vibration in the plane of the dissociated silanol molecule^35^ (Si–O–) potentially linked to another Si or H atom,^36^ associated with *Q* ^3^ silica species^37^ predominantly located in the microcavities of the zeolite.^38^ With increased milling time, the silanol band becomes more intense, suggesting an increase in the quantity of silanol groups with milling time.^39^ The shift in 1037 to 1051 cm^–1^ bands is presented; thus, it is more likely that grinding is causing elastic deformation in T–O–T bands.

**Figure 6:**
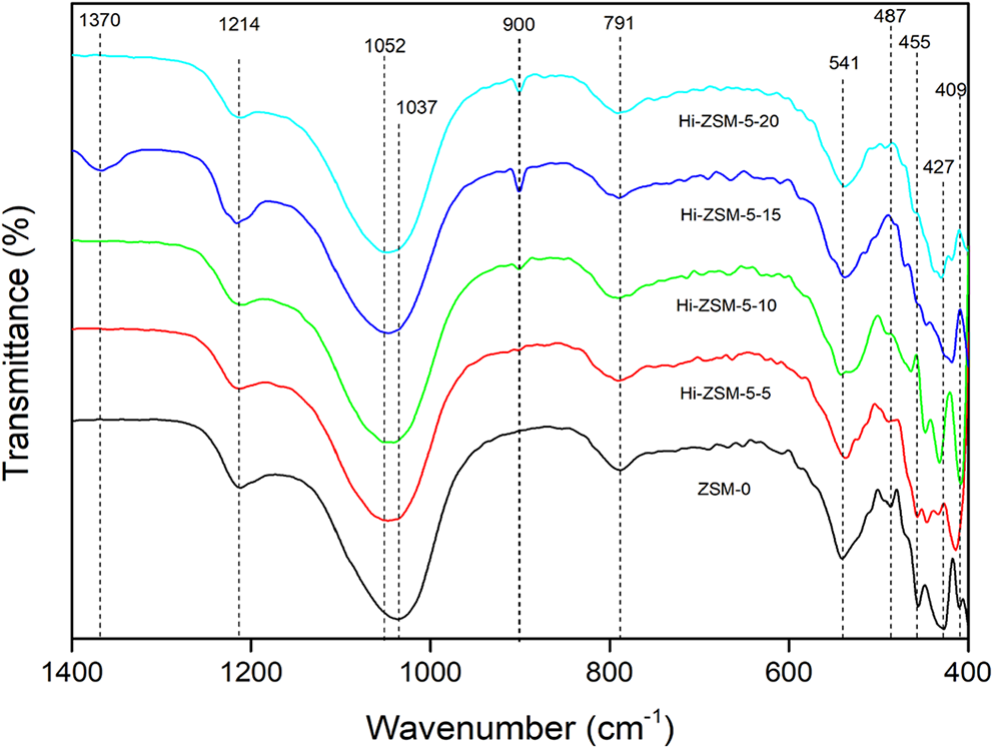
FTIR spectra of ZSM-5 zeolites with different milling times. The commercial zeolite corresponds to ZSM-5–0.

The characteristic 541 cm^–1^ band, which relates to the external T–O–T in D5R, remains present, but its intensity decreases proportionally with increasing grinding time. This decline suggests two concurrent effects: (i) a loss in crystallinity, which is independently evidenced by XRD data, and (ii) a significant disruption of the overall zeolite network.^40^ Furthermore, the decrease in the intensity of the 541 cm^–1^ band is recognized as the spectroscopic signature of the transition from intrinsic microporosity to induced mesoporosity through structural cleavage. The extent of this intensity decrease effectively serves to quantify the fraction of the original crystalline microporosity that has been converted into structural defects and mesoporous voids.^41,42^

The bands between 460 and 400 cm^–1^ represent internal SiO_4_ and AlO_4_ tetrahedron units^43^ and show significant variation during ball milling. The bands at 450 and 430 present in ZSM-5–0 are less intense after 5 min of milling, confirming that this time is sufficient to break the bonds of the five-membered ring and Si–O bending vibrations. After 10 min of milling, the bands are more intense than in ZSM-5–0, suggesting that mechanochemical treatment breaks Si–O bonds and restructures in the presence of CTMA, generating better chemical bonds and possibly mesopores. At 15 min, the band disappears and reappears at 20 min, showing that the process of bond breaking and recrystallization occurs during ball milling. The bands between 410 and 300 cm^–1^ emerge due to the natural movement of the zeolites^44^ and are present at 5 and 10 min of milling; nevertheless, they disappear after 15 and 20 min, suggesting that the bonds present in the micropores are being altered and possibly shifted to regions of higher energy.

These bands are highly sensitive to changes in crystallinity; consequently, a shift to a higher frequency is often associated with a decrease in the unit cell parameters in the tetrahedral sites. Furthermore, this type of shift can also be directly linked to the substitution of silicon (Si) by aluminum (Al) in the zeolite framework, which increases the proportion of Al–O bonds.^31,35^ The literature establishes a clear contrast between framework modes: intratetrahedral modes (950–1250 cm^–1^ and 420–500 cm^–1^) show only minor changes when the zeolite is partially destroyed (e.g., by thermal treatment), whereas intertetrahedral modes (500–650 cm^–1^ and 300–420 cm^–1^) decrease significantly in intensity or disappear entirely upon destruction.^45^

This distinction is crucial. If thermal treatment, a process often limited to surface effects, can cause such structural changes, then the sustained structural integrity combined with the observed chemical shifts strongly indicates a deeper modification. It demonstrates that mechanical activation is achieving more than just surface, elastic, and plastic deformation; it is enabling the templates to diffuse and induce a chemical reaction directly within the pores of the zeolite structure.

The presence of a band at 1370 cm^–1^ in the Hi-ZSM-5–15 spectrum is not attributable to the zeolite framework, suggesting the existence of an extra-framework species or residual contamination.^46^ This frequency strongly corresponds to the absorption of the nitrate ion (NO_3_^–^).^47^ It is hypothesized that the ion is a residual product formed during the N_2_ calcination step, where the organic CTMA^+^ template decomposes and releases nitrogen-containing species. Given the catalyst’s acid sites, the nitrate ion may be chemisorbed onto the pores, accounting for its detection after thermal treatment.

### 3.3 Scanning Electron Microscopy

The SEM images of the samples are shown in Figure 7. Figure 7A shows the untreated commercial zeolite, exhibiting microstructured crystallites with the characteristic cuboid shape, which is distinctive of the ZSM-5 structure and is characterized by well-defined regular square ridges with a flat surface extending across the entirety of the crystal.^48,49^ Figure 7B demonstrates that after 5 min of ball milling in the presence of TPA and CTMA, ZSM-5 is sufficient to reduce the overall size without undergoing amorphization as can be confirmed by the average particle size in Figure S6.

**Figure 7:**
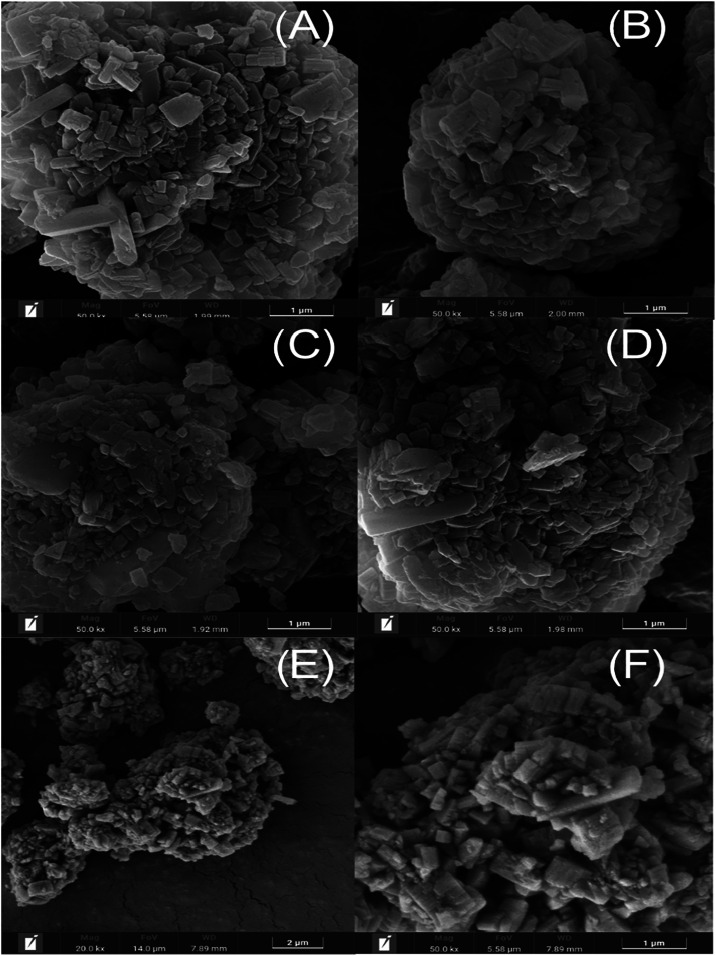
Scanning electron microscopy (SEM) images of commercial ZSM-5 (A) and mechanochemistry activated with cotemplates in 5 min of grinding (B) 10 min (C), 15 min (D), and 20 min (E, F).

Figure 7B–D shows samples treated with milling for 5, 10 and 15 min, respectively. They display a reduction in crystal size and fractures on the zeolite surface. This indicates that milling efficiently disrupted the morphology of ZSM-5, breaking chemical bonds in the surface without losing crystallinity. This suggests that the breakage of Si–O siloxane bonds forces CTMABr and TPABr to regroup, allowing for the rearrangement inside of the zeolite pores, possibly forming intercrystalline mesopores, as is well described in Figure S7 that presents the thermal behavior of the materials with mechanochemical activation.^35^

Figure 7(E,F) shows samples treated with milling for 20 min; they reduced the crystal size of HZSM-5 to small rectangular crystals with disordered spaces, closely resembling a combination of meso-macroporous structures in the form of nonuniform straight channels with nonsystematically ordered macropores, potentially arising from controlled combustion during calcination and the removal of organic templates.^50,51^

Therefore, the SEM images sustain the discussion presented using XRD and FTIR. The data confirm that mechanical activation supplies sufficient energy to initiate the cleavage of the siloxane bonds in the surface of the material and the subsequent reorganization of the structure through chemical reactions inside the pores. This combined process successfully maintains the integrity of the microporous framework, while introducing modifications to the interconnected porosity.

### 3.4 Nitrogen Adsorption and Desorption Properties

The N_2_ physisorption isotherms and pore size distributions are presented in Figure 8. The ZSM-5–0 (parent material), Hi-ZSM-5–5, and Hi-ZSM-5–20 samples exhibit Type II isotherms, which are characteristic of nonporous or macroporous solids, typically observed in conventional zeolites where crystal aggregation dominates the external surface behavior.^52^ The high adsorption at a low relative pressure confirms the intrinsic microporosity of the framework. In sharp contrast, the Hi-ZSM-5–10 and Hi-ZSM-5–15 samples display clear Type I­(b) isotherms. According to the IUPAC technical report,^53^ Type I­(b) is characteristic of molecular sieves with a relatively wide range of micropore sizes and small external surface areas, often indicating an initial stage of hierarchical porosity.

**Figure 8:**
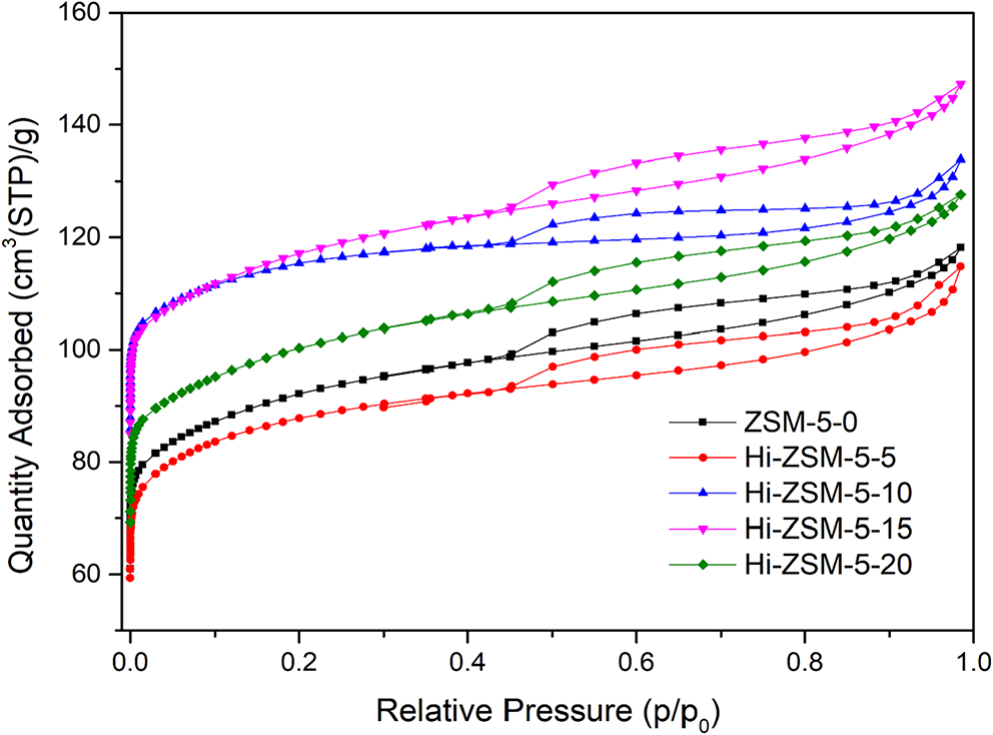
Nitrogen adsorption and desorption isotherms of zeolites activated by mechanochemical treatment at various milling times.

All five modified samples (Hi-ZSM-5) show the presence of a H4 hysteresis loop. The IUPAC (2015) explicitly correlates the H4 loop with capillary condensation in mesopores that are characteristic of slit-shaped pores or complex pore networks within aggregated materials, such as zeolites.^54^ The persistence of the H4 loop, coupled with the shift to a Type I­(b) isotherm in the optimally milled samples, strongly suggests two key effects of the mechanochemical treatment: The grinding promotes crystal agglomeration (as observed in SEM results), which increases the quantity of absorbed N_2_; the maintenance of the H4 loop confirms the successful creation of an interconnected mesoporous network alongside the intrinsic microporosity.

Table 4 describes the data from the textural analysis. The specific surface area of the commercial ZSM-5 (*S*_BET_) is 364 m^2^/g, and after just 5 min, there is a decrease of 29% in *S*_(BET)_, to 254 m^2^/g. The micropore area (*S*_micro_) also reduces by 37%, from 250 to 158 m^2^/g, with an 11% reduction in external surface area (*S*_ext_), from 114 to 101 m^2^/g. The micropore volume (*V*_micro_) decreases by 36%, from 0.10 cm^3^/g to 0.064 cm^3^/g; although, there is an 8% increase in mesopore volume (*V*_meso_), from 0.092 to 0.10, and a 6% decrease in pore diameter (*D*_p_), from 2.6 to 2.45 nm.

**Table 4:** Nitrogen Adsorption and Desorption of the ZSM-5 Mechanochemically Activated with Double Templating as a Function of Time

sample	*S*_BET_ (m^2^/g)^a^	*S*_micro_ (m^2^/g)^b^	*S*_ext_ (m^2^/g)^c^	*V*_micro_ (cm^3^/g)^d^	*V*_meso_ (cm^3^/g)^e^	*D*_p_ (nm)^f^	FH^g^
ZSM-5–0	364	250	114	0.10	0.092	2.6	0.164
Hi-ZSM-5–5	259	158	101	0.064	0.10	2.45	0.167
Hi-ZSM-5–10	387	290	98	0.11	0.072	2.38	0.155
Hi-ZSM-5–15	381	255	125	0.10	0.10	2.77	0.164
Hi-ZSM-5–20	379	265	115	0.10	0.10	2.7	0.161

a where *S*_BET_ is the BET surface area.

b *S*_micro_ is the micropore surface area evaluated by the *t*-Plot method.

c *S*_ext_ is the external surface area evaluated by the *t*-Plot method.

d *V*_micro_ is the micropore volume calculated by the *t*-plot method.

e *V*_meso_ is the volume of mesopores calculated by the BJH adsorption cumulative method between 10,000 nm and 300,0000 nm width.

f *D*_p_ is the BJH adsorption average pore width.

g FH is the hierarchical factor (*V*_micro_/*V*_total_) × (*S*_ext_/*S*_BET_).

Figure 9 presents the pore size distribution (PSD) of the parent and mechanochemically activated HZSM-5 catalysts, calculated for the 1 to 3 nm range using a pore size distribution Barrett–Joyner–Halenda (BJH) method derived from the N_2_ isotherm. All samples primarily display a very concentrated micropore size distribution. Nonetheless the Hi-ZSM-5–20 sample exhibits the lowest peak intensity, a characteristic often associated with the formation of well-defined mesopores and a wider pore diameter distribution.^55,56^ Conversely, the Hi-ZSM-5–5 sample presents a narrower pore width compared to the other samples, which may suggest the initial formation of mesopores influenced by the grinding process followed by the thermal decomposition of the CTMA template at high temperatures.^57^

**Figure 9:**
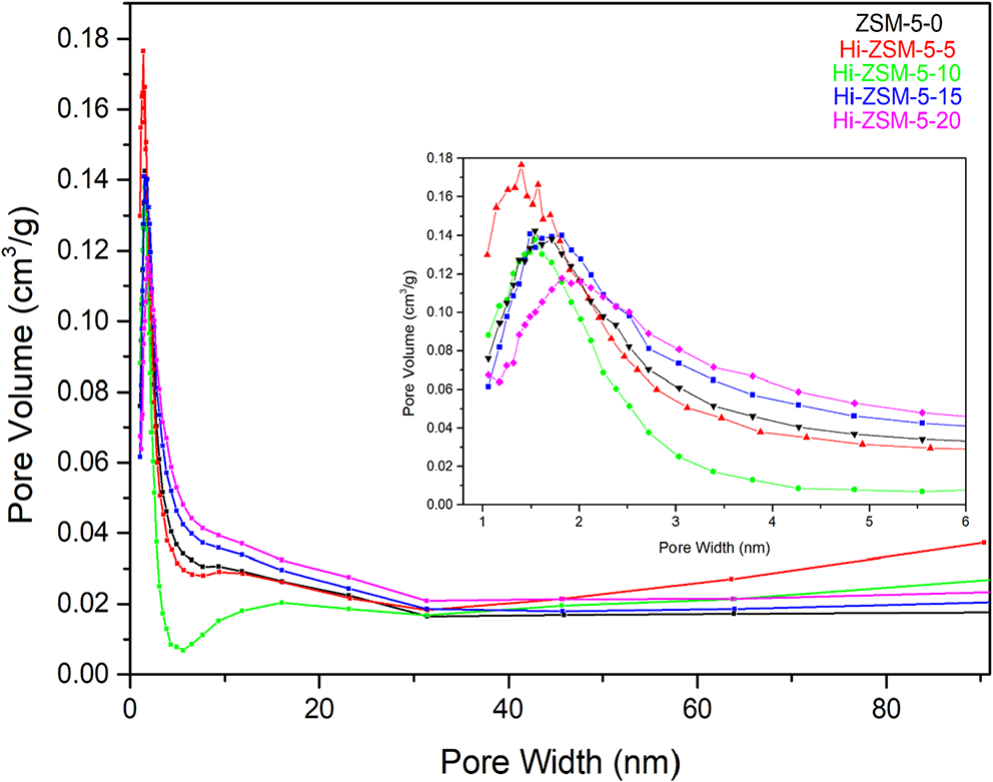
Pore size distribution of zeolites is activated by mechanochemical treatment at various milling times.

Further analysis of the N_2_ isotherm for Hi-ZSM-5–5 reveals that the pore volume increases linearly with the increase in the relative pressure (*p*/*p*_0_) up to the saturation point. This linear rise is typically attributed to the stacking of crystalline grains and the presence of macropores.^58,59^ While confirming the existence of macropores in this specific material requires further characterization (e.g., mercury porosimetry), SEM images visually support this finding. The micrographs clearly show large void spaces, which are attributed to crystal stacking and aggregation induced by the milling process and are consistent with the presence of macroporosity.

The data are consistent with the reduction in crystallinity and crystal size, indicating that milling provided sufficient energy in just 5 min to cause modifications directly on the surface and pores of the structure. During the milling process, the templates were responsible for recrystallizing, restoring,^35^ and restructuring the sample, slightly increasing the pore size. However, the restructuring was not accompanied by a large widening. After 10 min of milling, there was an increase of 6% in *S*_BET_, 14% in *S*_micro_, 10% in *V*_micro_, and 8% in *V*_meso_, a decrease of 14% in S_ext_ and 8.5% in *D*_p_ to 2.38 nm. Fifteen minutes of grinding has the higher increase in *S*_ext_ area of 9% 114 to 125 cm^3^/g and higher increase in 6̈% *D*_P_ 2.6 to 2.77 nm. After 20 min, there was a similar restructuring of textural properties, with a 4.0% increase in *S*_BET_, 6% in *S*_micro_, 8.8% in *S*_ext_, and 8% in *V*_meso_, with *D*_p_ increasing 4%, indicating that CTMA^51^ is responsible for recrystallizing the micropores destroyed after 5 min of milling.

The primary objective of employing SDA during ball milling was to use mechanical energy to induce a chemical reaction within the zeolite’s porous structure, facilitating a structure-to-structure (S-T) transformation to form mesopores while avoiding the generation of a significant amorphous phase.^60^ This avoidance is primarily attributed to the short grinding duration used. While TPA^+^ is generally known to have a silicon protective effect due to its low alkalinity, which prevents silicon removal and can even repair the crystalline structure,^61^ the high energy supplied by the grinding procedure appears sufficient to override this protective role and induce desilication. This structural change is directly evidenced by the decrease in the Si/Al ratio observed in the Supporting Information (SI. Table S1).

This chemical modification is corroborated by multiple characterization techniques. The IR spectral shifts, specifically the appearance of a band at 900 cm^–1^ and the corresponding change in the 455 cm^–1^ band, are consistent with the breaking and reforming of Si–O–Si (silanol) bonds within the microcavities. Furthermore, the concurrent increase and subsequent decrease in the surface area, pore volume, and pore diameter (summarized in Table 4) strongly suggest that the ball mill supplies enough energy to drive a dynamic equilibrium of bond cleavage and formation, likely through the generation of transient radicals during grinding. The fact that we did not observe a substantial increase in total mesopore volume is attributed to the simultaneous recrystallization process regenerating the material. Collectively, this evidence, along with the previous characterization, demonstrates that ball milling with dual SDA successfully modified the porosity and channeling structure of the ZSM–5 zeolite.^62^

The development of mesopores must be accompanied by the preservation of microporosity; otherwise, the materials would become excessively mesoporous or microporous. As a result, hierarchical porosity can be evaluated using the Hierarchical Factor (HF); low FH values tend to be on one extreme, corresponding to micropores, while high FH values are caused by the presence of mesopores; therefore, the range of hierarchical zeolite lies between both. To be recognized with hierarchical porosity, the material needs to present the microporosity characteristic of the initial material with interconnect mesoporous in the structural microporosity channel, that means an intermediate value of FH.^63,64^ Sure, this sentence is open to discussion, once the FH just indicates the characteristics of the material and does not give accurate information about interconnection between pores.^65,66^ The following formula was used to calculate it: HF = (*V*_micro_/*V*_total_) × (*S*_ext_/*S*_BET_). The calculated values for the commercial zeolite, ZSM-5–0, were 0.165, and for 5, 10, 15, and 20 min of milling, the values corresponded to 0.167, 0.155, 0.164, and 0.161, respectively.

The mechanochemical process did not result in a simple enlargement of the intrinsic micropores. While the literature^18,22^ generally reports a significant decrease in micropore volume and a substantial increase in mesopore volume (by a factor of 2 to three), our work achieved a 10% increase in mesopore volume while successfully maintaining the intrinsic microporosity. This result does not imply the absence of hierarchical porosity. Instead, the modification mechanism primarily involved the SDA acting as highly reactive radicals during high energy milling, rather than interacting as conventional bulky molecular ions.^54^ This radical-based interaction led to the formation of strong chemical bonds between the template fragments and the silanol sites, successfully altering the pore distribution and indicating deep modification within the zeolite’s porosity.

Specifically, TPA^+^ species on the catalyst surface are believed to be responsible for the initial cleavage of silanol bonds (Si–O–Si) driven by the energy provided by grinding.^17^ Conversely, the CTMA^+^ is theorized to contribute to the formation of new Si–O–Si bonds within the porous channels via the structure-to-structure (S-T) mechanism.^51^ This dynamic balance between bond cleavage and structural regeneration is corroborated by the maintenance of relative crystallinity and crystallite size, alongside the distinct phenomena of appearing/disappearing and creation of new infrared bands. Collectively, these morphological and spectroscopic findings strongly suggest that the mechanochemical treatment with dual SDA successfully induced structural features consistent with a hierarchical ZSM–5 catalyst.

### 3.5 Total Acidity through Thermal Desorption of the *n*-Butylamine Method

Figure 9 and Table 5 show the method of *n*-butylamine adsorption and thermal desorption by TG for Hi-ZSM-5–20, used to determine the density of the acidic sites. Since each mole of *n*-butylamine is adsorbed onto one mole of acidic sites, the acidity was calculated.^67^ The thermogravimetric results revealed the profile of acidic sites during the breakdown of *n*-butylamine. The second and third stages, in the temperature ranges of 100–300 °C and 300–640 °C, respectively, were attributed to n-butylamine chemisorbed on medium and strong acidic sites.^68^ The first temperature range, observed between 30 and 100 °C, was assigned to physisorption and the loss of *n*-butylamine adsorbed on weak acidic sites. Additionally, the density of acidic sites can be calculated by using mass loss.

**Table 5:** Concentration of Acidic Sites through Thermal Desorption of *n*-Butylamine in the Catalyst with Mechanochemistry Activation under Optimized Ball Mill Conditions after 20 Minutes of Milling

	concentration of the acidic sites (mmol/g)		
sample	I -Brønsted sites (100–300 °C)	II - lewis sites (300–900 °C)	total acidity
Hi-ZSM-5–20	0.62	1.23	1.85

The reaction between n-butylamine and protonated amine was associated with the DTG peak (Figure 9) in the temperature range of 600–880 °C, based on the reaction described below.^69,70^ The formation of butene, ammonia, and diamine on acidic zeolites has been demonstrated by other researchers using NMR,^69^ infrared spectroscopy,^71^ and mass spectrometry.^72,73^

A higher total acidity of 1.85 mmol/g was observed compared to the commercial zeolite studied with 1.52 mmol/g, and the template-free synthesized zeolite with 1.54 mmol/g reported by ref 73. The strength of the strong acidic sites is associated with Bronsted protonic acidity, as it requires more energy to break the interaction between the adsorbed base and the protons. Also, higher than most recent work^74^ developed several hierarchical ZSM-5 zeolites, and the highest concentration of the total acidity sites was 1.14 mmol/g.

The acidity sites are related to the surface area of the material, the acidity increases with the increase of the surface as confirmed on textural analysis, and the presence of the aluminum also can vary the acidity of the material, so the Si/Al of the materials compared before should be taken in place rather than the analysis in fact.^75^ Also, mechanochemical activation does not contribute to reducing the aluminum in the structure as confirmed.^76^ Thus, the increase in the acidity of the material is attributed to mechanochemical activation (Figure 10).

**Figure 10:**
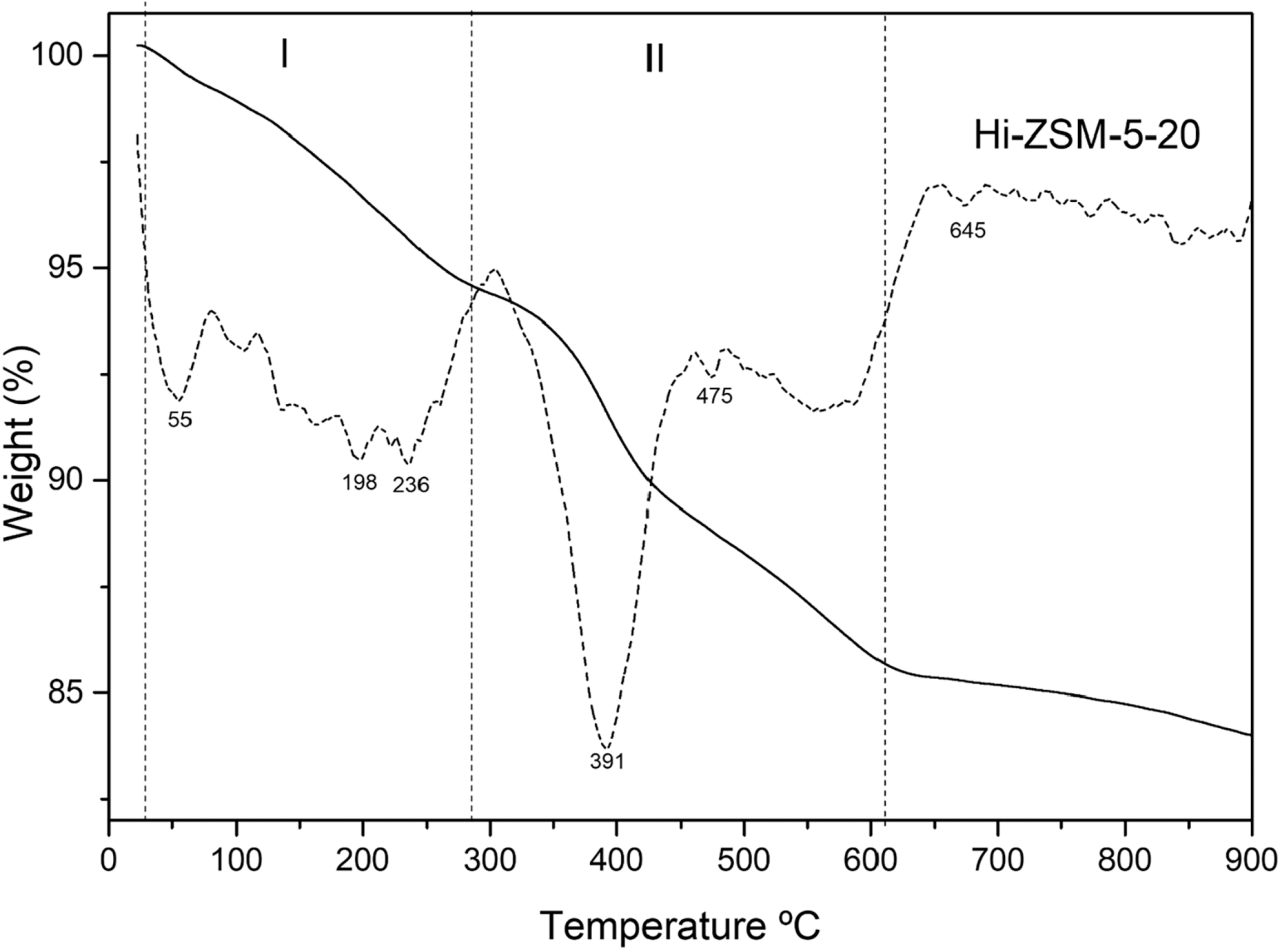
Thermal behavior of Hi-ZSM-5–20 with adsorbed *n*-butylamine. I represents the medium sites desorption and II the strong sites.

Also, it is important to mention that there is a severe desorption in high temperature, this is related to the coke formatted inside the microcavities through the mechanical action between zeolite and templates; the same behavior is presented in S.I. for the thermal decomposition of zeolites with templates (Figure S7 and Table S2). The presence of coke is supported by the slight shifts in XRD data, EDS carbon peaks presented (Figures S1 and S2) and are summarized in Table S1.

### 3.6 Thermocatalytic Pyrolysis of Polypropylene with Mechanochemically Activated ZSM-5

Pyrolysis of PP pellets, both in the presence and absence of a catalyst, resulted in the formation of three product fractions: condensable gases (collected as liquid oil), noncondensable gases, and a solid product (coke). Given the storage constraints, all noncondensable gases were necessarily vented following cooling. Thus, the analysis was limited to the liquid content via GC-MS. Figures S3–S5 display the chromatograms, which show the retention times of the pyrolysis products from PP, PP + ZSM-5–0, and PP + ZSM-5–20. Despite the uncollected nature of the noncondensable fraction, the experimental data show that 100% of the initial plastic mass was converted into products in both the thermal and thermocatalytic processes, as illustrated in Figure 11A.

**Figure 11:**
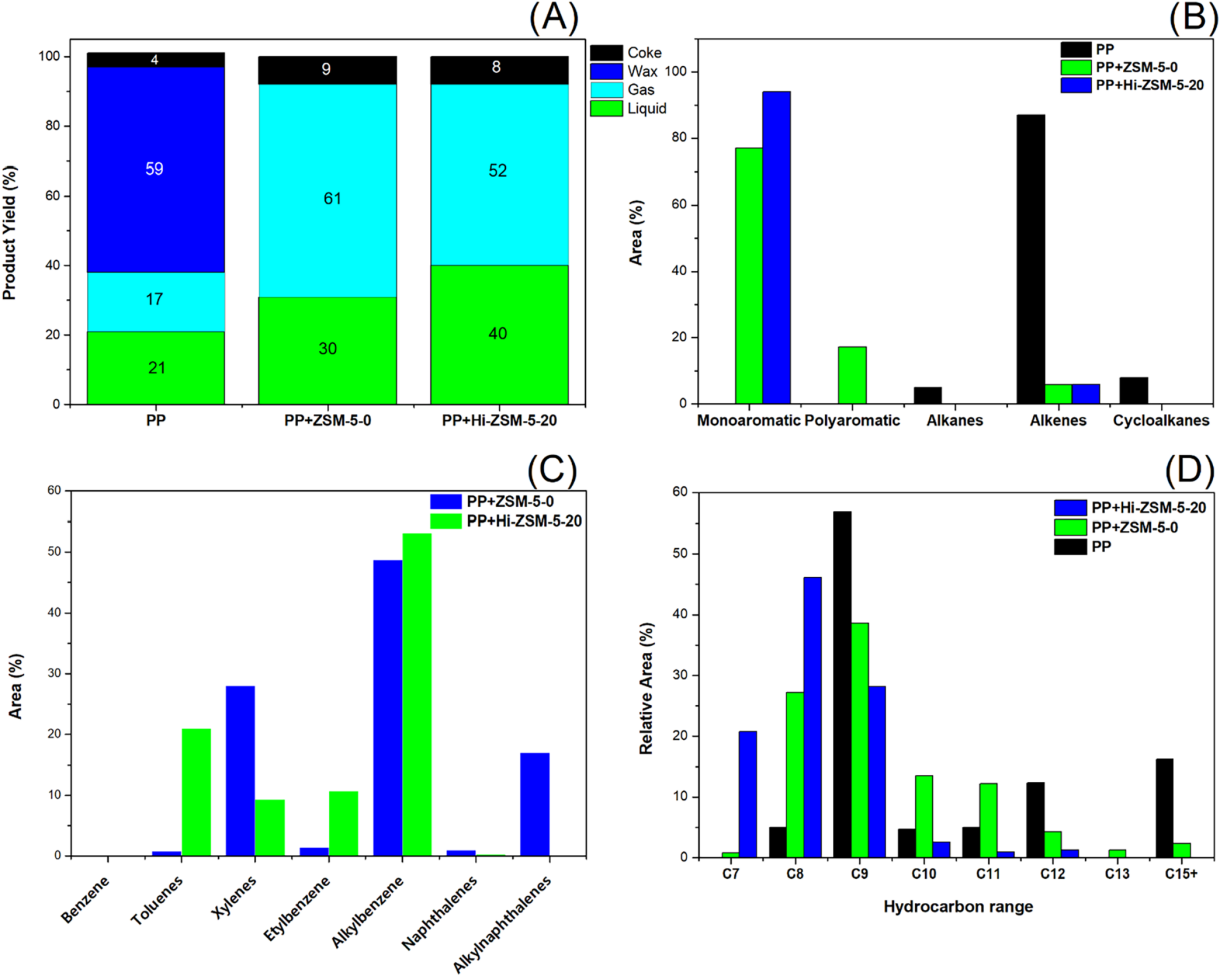
Catalyst test of the pyrolysis of polypropylene without catalyst, commercial ZSM-5, and mechanochemistry activated ZSM-5: (A) Yield in weight %; (B) major products; (C) major individual compounds in the catalytic pyrolysis; (D) hydrocarbon range

The yield for PP pyrolysis was 59% wax, 20% oil, 17% gas, and 4% coke, whereas in thermocatalytic pyrolysis, the use of the ZSM-5 catalyst with a low mesopore volume favored gas formation by facilitating the retention of radicals within the zeolite pores during the initiation and propagation stages of the reaction. This prolonged cracking of large PP molecules hinders the release of large molecules and justifies the 61.2% increase in gas generation. It is proposed that the presence of the catalyst is responsible for this behavior, while plastic retained on the catalyst’s surface favored repolymerization, leading to an 8.1% increase in coke formation, despite the relatively small fraction of oil produced at 30.7%; nevertheless, there is no wax formation during the catalyst pyrolysis.

The resulting oil was a slightly yellowish liquid, whereas the light and dense oil at room temperature corresponded to a yellowish solid, indicating that the formed oil contains light hydrocarbons within the range of biofuels.^77,78^ Thermocatalytic pyrolysis of PP with ball-milled catalyst modification exhibited superior performance in producing light oil, with no dense phase formation and a 40% yield of oil and a decrease in the gases, 52% and 8% coke; the modification in the pore structure during the ball milling showed better improvement in the catalyst pyrolysis.

Figure 11B shows major products of the cracking of PP and catalyst cracking. The cracking of PP was more selective toward olefins, pure PP pyrolysis was incapable of forming monoaromatic hydrocarbons but was selective for olefins, accounting for 87% of the yield, alongside the concurrent formation of alkanes and alkenes. the primary products of PP pellet pyrolysis are 2,3-dimethyl-2-heptene, 2,4-dimethyl-1-heptene, 2,4-dimethyl-1-decene, 2,2-dimethyl-3-octene, and (*E*)-3-eicosene. It also generated 5% alkanes, predominantly 4-methylheptane, and 8% cycloalkanes, 1,3,5-trimethylcyclohexane, corresponding to the gasoline, naphtha, kerosene, and diesel range. The presence of more aliphatic hydrocarbons in the PP pyrolysis oil suggests that weaker secondary reactions to cyclization occurred during the cracking process.^79,80^ Catalytic pyrolysis of PP using the commercial ZSM-5 zeolite primarily produced 77% monoaromatic compounds as well as alkenes 6% and 17% polyaromatics. The results are in accordance with literature that suggests that HZSM-5 zeolite micropores specifically accelerate the synthesis of aromatic compounds due to the olefins present during polypropylene cracking.^81^ Catalytic pyrolysis of PP using ball-milled modified zeolite demonstrated superiority in terms of aromatic compound formation, with 94% of the green diesel yield collected in the flask consisting of BTEX’s compounds, while the remaining 6% consisted of olefins.

Figure 11C shows individual compounds in the catalyst pyrolysis of PP with commercial ZSM-5 and with activated ZSM-5. Commercial zeolite was formed in a major manner of 28% *p*-xylene, 48.8% alkylbenzene, and 17% alkylnaphthalene, and mechanochemically activated ZSM-5 was more selective to toluene, ethylbenzene, and alkylbenzene. It is recognized that the acidity and pore structure of ZSM-5 zeolite significantly affect catalytic cracking performance^82^ and the activated ZSM-5 has better acidity compared with other synthesized zeolites with similar SAR. Also, when the mechanochemistry modified ZSM-5 was applied, it reduced all naphthalene, being more selective to BTEX’s compounds such as 21% toluene, 9.3% p-xylene, 10.7% ethylbenzene, and 53.2% of alkylbenzenes such as 27.2% 1,1-diethylbenzene, 15.5% 1-ethyl-3-methyl-benzene, 7.9% 1-ethyl-2-methyl-benzene, and 2,6 1,3-diethyl-benzene. This indicates selectivity for monoaromatic compounds with reduced polyaromatics compared to pyrolysis using commercial zeolite owing to the favoring of Diels–Alder reactions. The mechanically modified catalyst (Hi-ZSM-5–20) had a more significant impact on the selectivity toward monoaromatic compounds due to its pore distribution being comparable to the kinetic diameter of aromatic compounds. Through the Diels–Alder reaction, the cracked gas olefins were transformed to aromatic hydrocarbons. In oligomerization, benzene, toluene, and other alkyl aromatics were produced by breaking down long-chain hydrocarbons from light olefins. In addition to monocyclic aromatic hydrocarbons, the Diels–Alder reaction also has the capacity to produce naphthalene and its derivatives.^83^

Figure 11D sows the hydrocarbon range of the pyrolysis oil products. Pure PP pyrolysis presents a hydrocarbon range C_8_–*C*_20_ which represents the range of gasoline, diesel, and kerosene; the catalyst pyrolysis with commercial ZSM-5 results in C_7_–C_15_; due to the naphthalene content, there no significant application of the catalyst as a fuel, but the mechanochemistry activated ZSM-5 formed a monoaromatic range C_7_–C_9_ mainly containing BTEX’s compounds which are blended up to 20 vol% in the fuel system to improve elastomeric sealing properties and boost fuel density.^84^

A significant study involving ZSM–5 was conducted by Hongchang et al., who performed catalytic pyrolysis of PP using ZSM–5 with different Si/Al ratios (27, 80, and 150) both with and without the presence of the organic precursor microcrystalline cellulose (CMC), yielding six types of micromesoporous ZSM–5 catalysts.^81^ All catalysts were evaluated via Py-GC/MS. The highest yield for pyrolysis oil (32.1%) and the highest yield of BTEX compounds (79.7%) were achieved with the ZSM–5 having the lowest Si/Al ratio (27) in the presence of CMC.^85^ In comparison, the mechanochemically modified ZSM–5 catalyst (Si/Al = 13) developed in this work significantly outperformed the reported optimum catalyst. Our material yielded a higher fraction of the liquid oil (40%) and achieved notably superior selectivity toward BTEX compounds (94%). This demonstrates that ball-milled modification is an effective strategy for producing an improved hierarchical catalyst for the polypropylene pyrolysis process.

Ratnasari et al.^86^ investigated the pyrolysis of high-density polyethylene (HDPE) using a two-stage approach. They employed a mesoporous material (MCM–41) in the first stage to maximize the oil yield (83%) and a microporous material (HZSM–5) in the second stage to enhance aromatic selectivity (95%). A similar strategy, using a 1:1 mixture of both catalysts in a single stage, was also explored. They noted that their activated ZSM–5 showed good aromatic selectivity but, despite having better diffusion than commercial ZSM–5, was less efficient in oil yield compared to the mesoporous material. A similar concept was recently explored,^57^ which developed a ZSM-5@SBA-15 composite to enhance light aromatic products during polypropylene (PP) pyrolysis. Their composite catalyst achieved a 53% liquid yield, representing a 5% increase compared with the 48% yield obtained with pure ZSM–5. Crucially, the composite catalyst significantly reduced the number of heavy components (by 42%), resulting in a light aromatic compound concentration of approximately 50%.

The key difference between these references and our material lies in the mechanochemically activated ZSM–5, which possesses a higher interconnect porosity while essentially maintaining the microporous structure of the framework. While He et al.’s composite enhanced liquid yield by 5% by utilizing the mesoporous SBA–15 shell for initial precracking of bulky paraffins, the SBA–15 shell has few active sites. It relies on the underlying ZSM–5 core for the catalytic sites that favor aromatic formation. In contrast, our activated ZSM–5 achieved a 10% increase in liquid yield (totaling 40% liquid yield) despite remaining primarily microporous. This enhanced liquid production is directly attributed to the creation of hierarchical channels and the increased interconnectivity achieved through mechanochemical activation, surpassing the performance of commercial ZSM–5 without sacrificing the high active site density necessary for efficient aromatic conversion.

## 4 Conclusions

Zeolites ZSM-5 with MFI structure were subjected to mechanochemical treatment in the presence of CTMA and TPA dual directing agents, varying the time from 5 to 20 min, under rotation in a ball mill, aiming to obtain hierarchical zeolites. The materials were characterized by XRD, SEM, FTIR, and N_2_ adsorption–desorption. It was verified that the mechanochemical activation followed by recrystallization in the MFI morphology was not adequate to increase the size of the micropores; however, during the restructuring, a change in the conformation of the micropores is proposed and consequently new interactions between the ZSM-5 materials and the raw templates.

It was observed in the mechanochemical treatment the decrease in the particle size of ZSM-5 after 5 min of milling without amorphization of the crystalline structure. At longer times, an increase in the crystal size was observed during the treatment, resulting from the reactivity of silanol groups resulting from the mechanochemical treatment, with subsequent breaking of the five-membered ring, followed by recrystallization with CTMA. This is an indication that the treatment was efficient in promoting small breaks in –Si–O–Si– bonds on the surface and in the bonds of ZSM-5 without loss in the crystalline structure, favoring restructuring, recrystallization, and new chemical bonds with cotemplates TPA and CTMA, restructuring the pores of ZSM-5 containing restructured micropores and possibly modified due to recrystallization after mechanochemical treatment.

The modified mechanochemistry ZSM-5 has been applied in the catalyst pyrolysis of polypropylene to comprehend the differences between the activated ZSM-5 and commercial one; modified catalyst presented high selectivity to BTEX’s compounds, improved 77% to 94% yield and a 10% increase in the liquid yield by pyrolysis, also reduced all polyaromatics content, producing a liquid that can be performed as a sustainable additive to the aviation of kerosene fuel. The Hi-ZSM-5–20 showed the highest accessible active sites and better pore configuration that influences the pyrolysis of plastic.

## Supplementary Material



## Notes

Energy-dispersive X-ray spectroscopy (EDS), elemental mapping images, and EDS spectra of the samples: mechanical activated Hi-ZSM-5–10 and Hi-ZSM-5–20; Si/Al ratio calculated by integrating peaks in EDS spectra; chromatogram of the pyrolysis liquid of PP, PP+ZSM-5–0, and PP+ZSM-5–20; thermal behavior of ZSM-5 zeolite with ball mill treatment with and without SDA under optimal conditions; summary of the temperature range and weight loss of the grinded zeolites (PDF)
