# Supplementary material for: Physicochemical Properties of Mechanochemical Activated HZSM‑5 Zeolite for Thermocatalytic Pyrolysis of Polypropylene
Source: ACS Omega. 2025 Dec 26;11(1):861–76. doi: 10.1021/acsomega.5c07513 (PMC12809516; doi:10.1021/acsomega.5c07513)
Supplement: Supplementary file 1 [file ao5c07513_si_001.pdf]

Supporting Information

Physicochemical Properties of Mechanochemical Activated HZSM-5 Zeolite for Thermocatalytic Pyrolysis of Polypropylene

Pedro F.A.C. Queiroz[1,2]*, Marcio D.S. Araujo[1], Edjane F.B. Silva[2], Aruzza M.M. Araujo[2], Amanda D. Gondim[2], Valter J. Fernandes Jr.[3], Antonio S. Araujo[3,]*

[1] *Post-Graduate Program in Chemistry, Institute of Chemistry, Federal University of Rio Grande do Norte, Natal RN, 59078-970, Brazil.*

[2] *Laboratory of Environmental Analysis, Primary Processing and Biofuels, Institute of Chemistry, Federal University of Rio Grande do Norte. Natal RN, 59078-970, Brazil.*

[3] *Laboratory of Catalysis and Petrochemistry, Federal University of Rio Grande do Norte, Natal RN, 59078-970, Brazil.*

(*) Corresponding authors: pedro.queiroz.103@ufrn.edu.br (P.A.F.C.Q.)
antonio.araujo@ufrn.br (A.S.A.)

Fig. S1 Energy-Dispersive X-ray Spectroscopy (EDS) elemental mapping images and EDS spectra of the sample: mechanical activated Hi-ZSM-5-10.

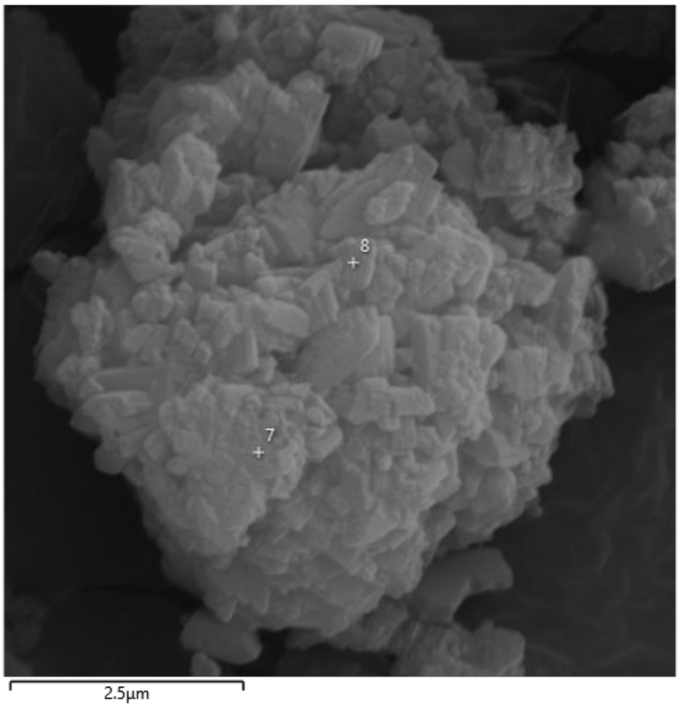

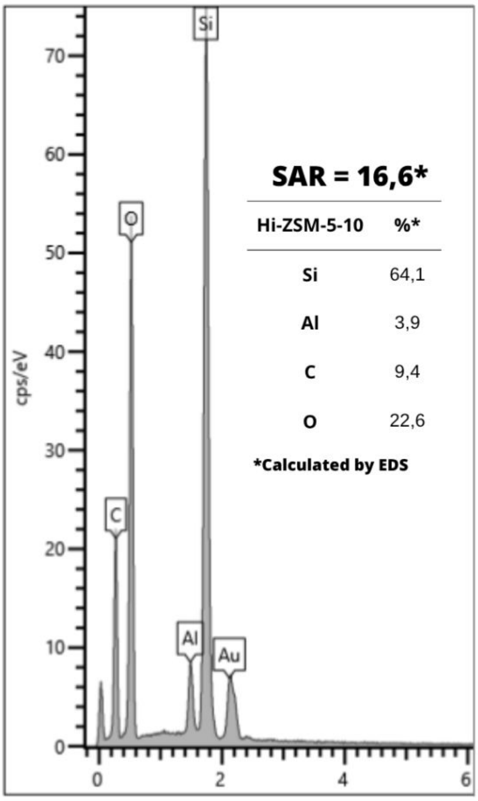

Fig. S2. EDS elemental mapping images and EDS spectra of the sample: mechanical activated Hi-ZSM-5-20.

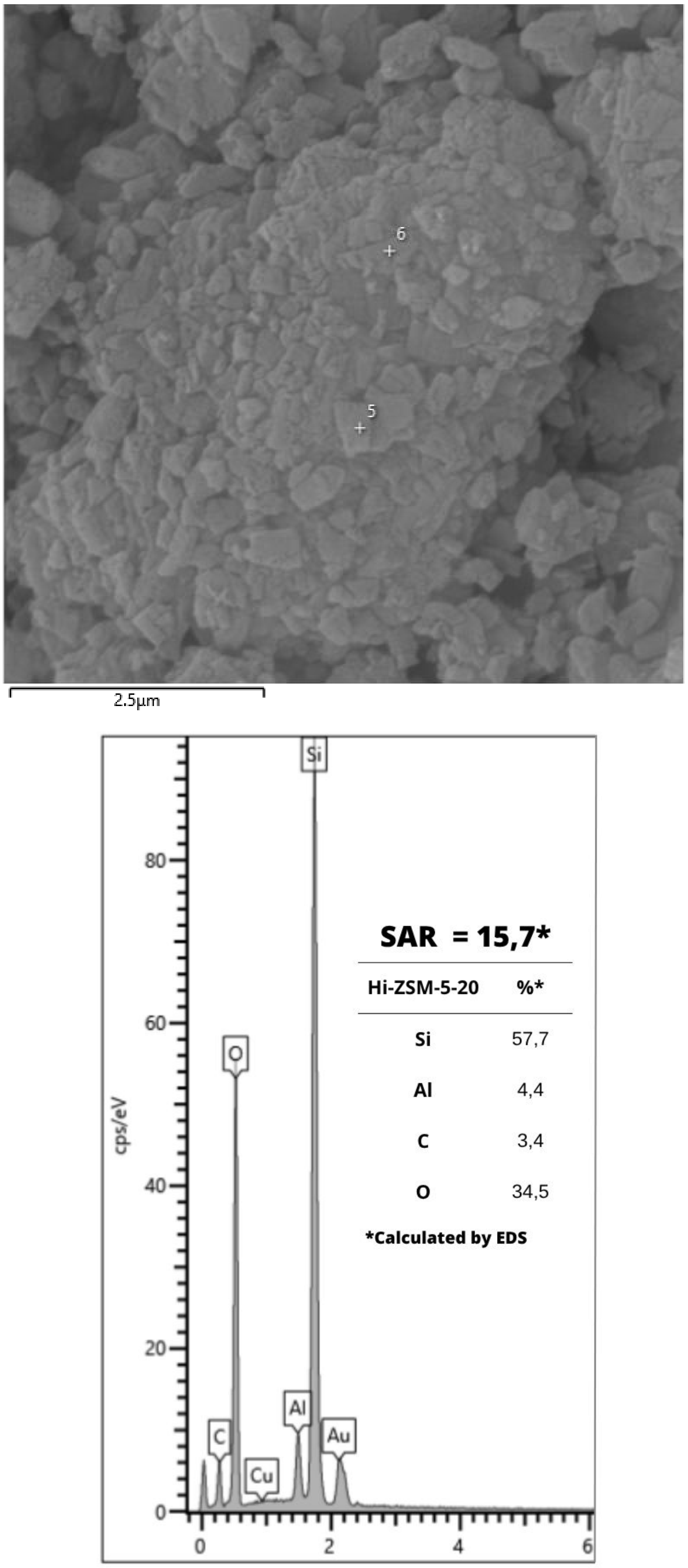

Table S1 Silicon and Aluminium ratio calculated by integrating the peak area of EDS spectra.

Sample	SAR
ZSM-5-0	23[a]
Hi-ZSM-5-10	16.6[b]
Hi-ZSM-5-20	15.7[a]

[a] provided by Zeolyst, [b] calculated by EDS

Fig. S3: Chromatogram of products obtained from the pyrolysis of PP pellets.

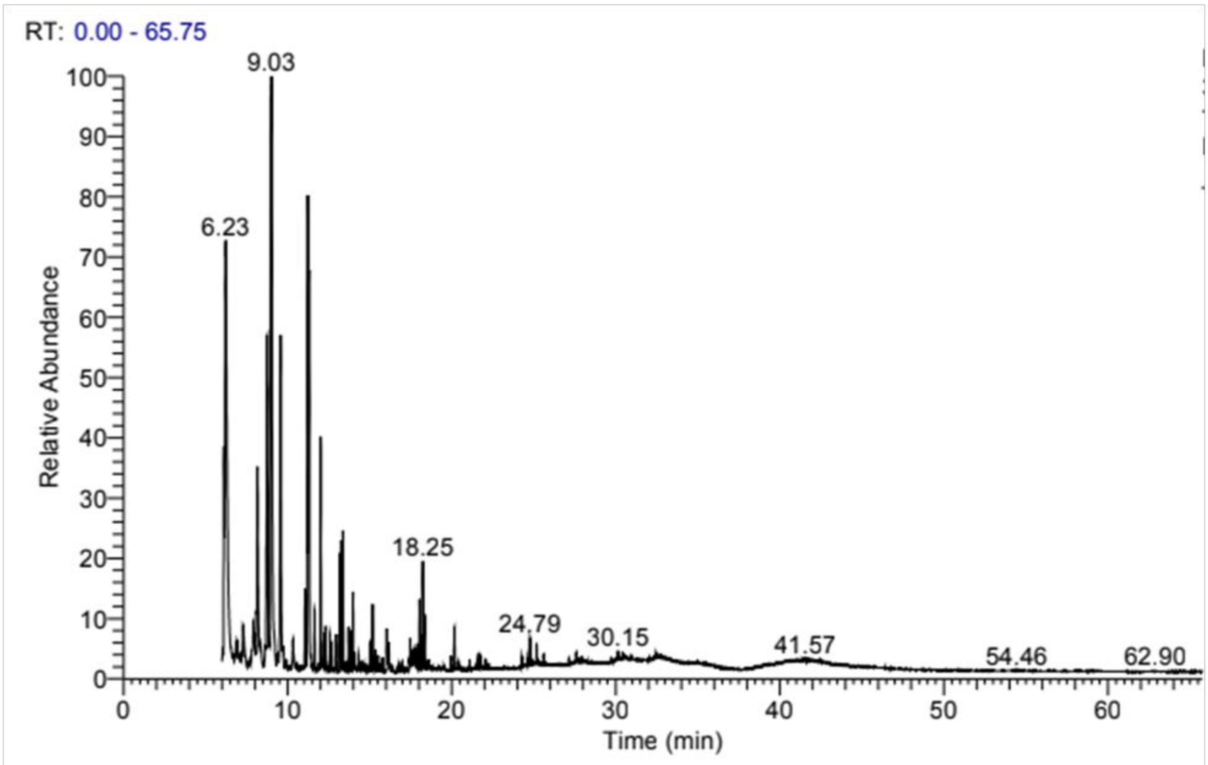

RT	Peak Area	Area %
6.13	2625815488.36	3.89
6.23	11414002876.45	16.92
8.16	4082890688.07	6.05
8.76	7208020279.15	10.69
9.02	18320242360.16	27.16
9.57	6268709441.61	9.29
11.24	7324736620.69	10.86
11.32	5328248525.34	7.90
12.01	3135715012.35	4.65
13.38	1735843284.51	2.57

Fig. S4.: Chromatogram of the pyrolysis products of PP pellets with commercial ZSM-5 (ZSM-5-0).

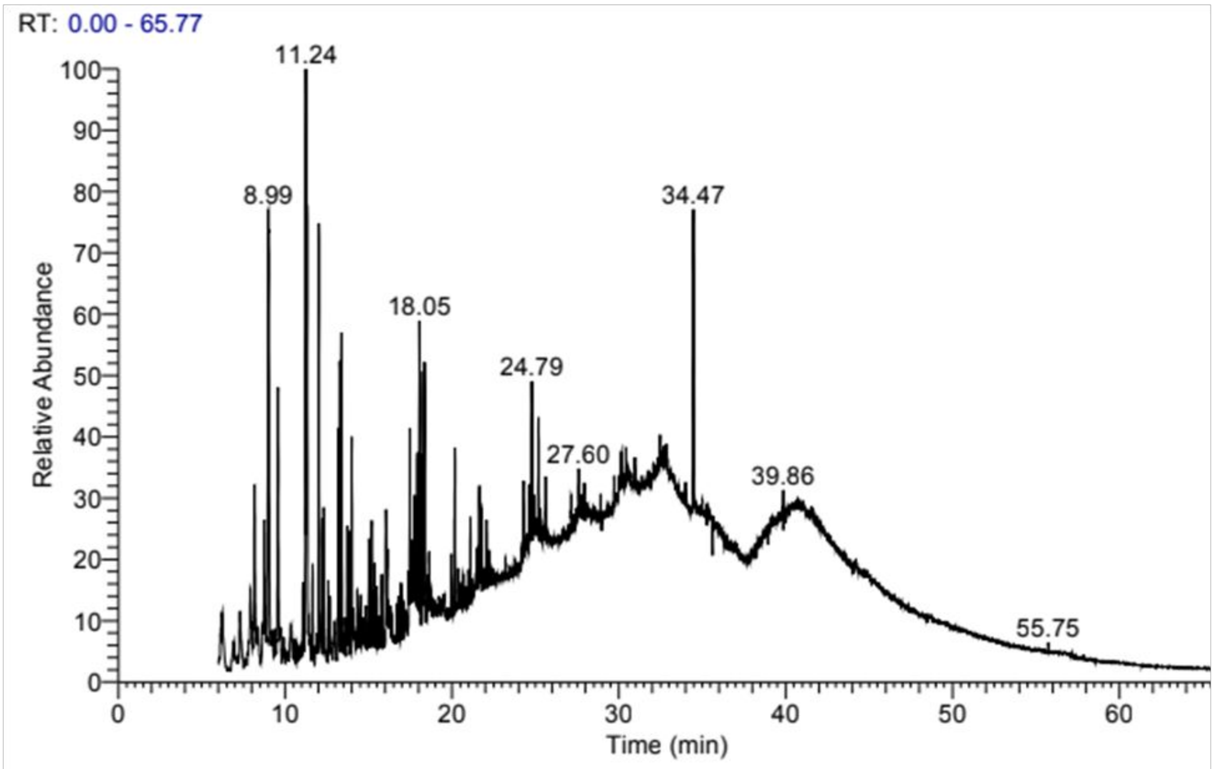

RT	Peak Area	Area %
8.16	2570856145.54	5.82
8.99	8904264100.07	20.15
9.57	3564171614.09	8.06
11.24	6937014677.98	15.70
11.32	4209241688.99	9.52
12.01	4653109783.96	10.53
13.27	2716626893.24	6.15
13.38	3204701746.91	7.25
18.26	3705307667.09	8.38
34.47	3733538582.10	8.45

Fig. S5. Chromatogram of PP pyrolysis products in pellets with ZSM-5 modified by mechanochemical activation (Hi-ZSM-5-20).

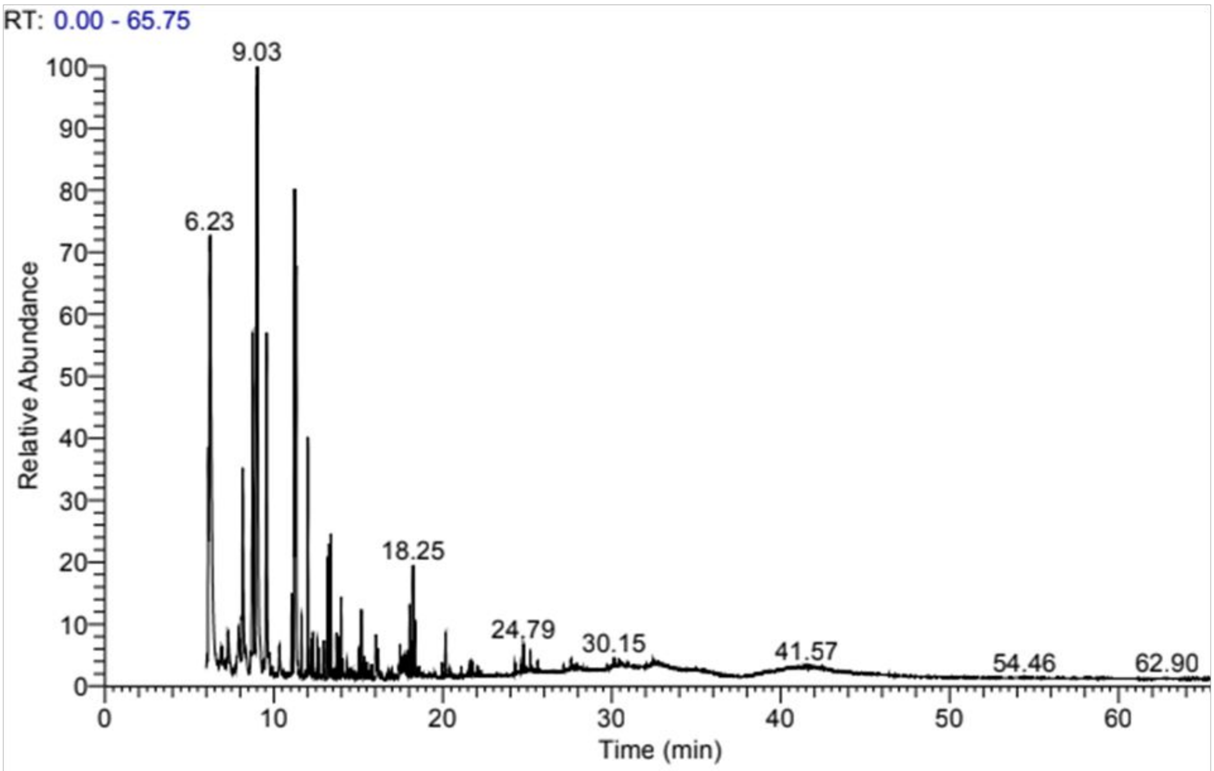

RT	Peak Area	Area %
6.13	2625815488.36	3.89
6.23	11414002876.45	16.92
8.16	4082890688.07	6.05
8.76	7208020279.15	10.69
9.02	18320242360.16	27.16
9.57	6268709441.61	9.29
11.24	7324736620.69	10.86
11.32	5328248525.34	7.90
12.01	3135715012.35	4.65
13.38	1735843284.51	2.57

Fig. S6. SEM and particle size distribution A) commercial ZSM-5, B) Hi-ZSM-5-5, C) Hi-ZSM-5-10, D) Hi-ZSM-5-15 and E) Hi-ZSM-5-20 samples.

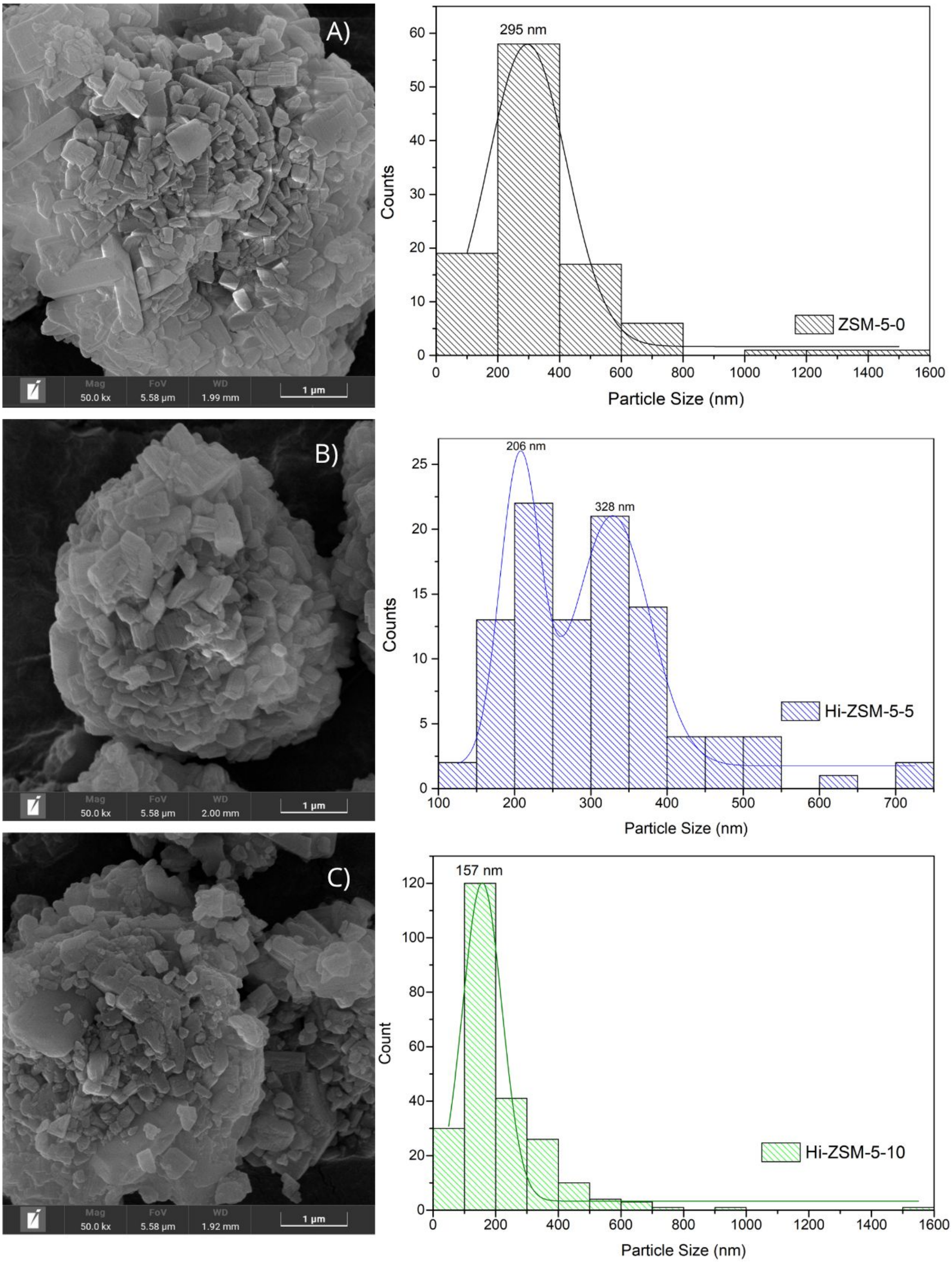

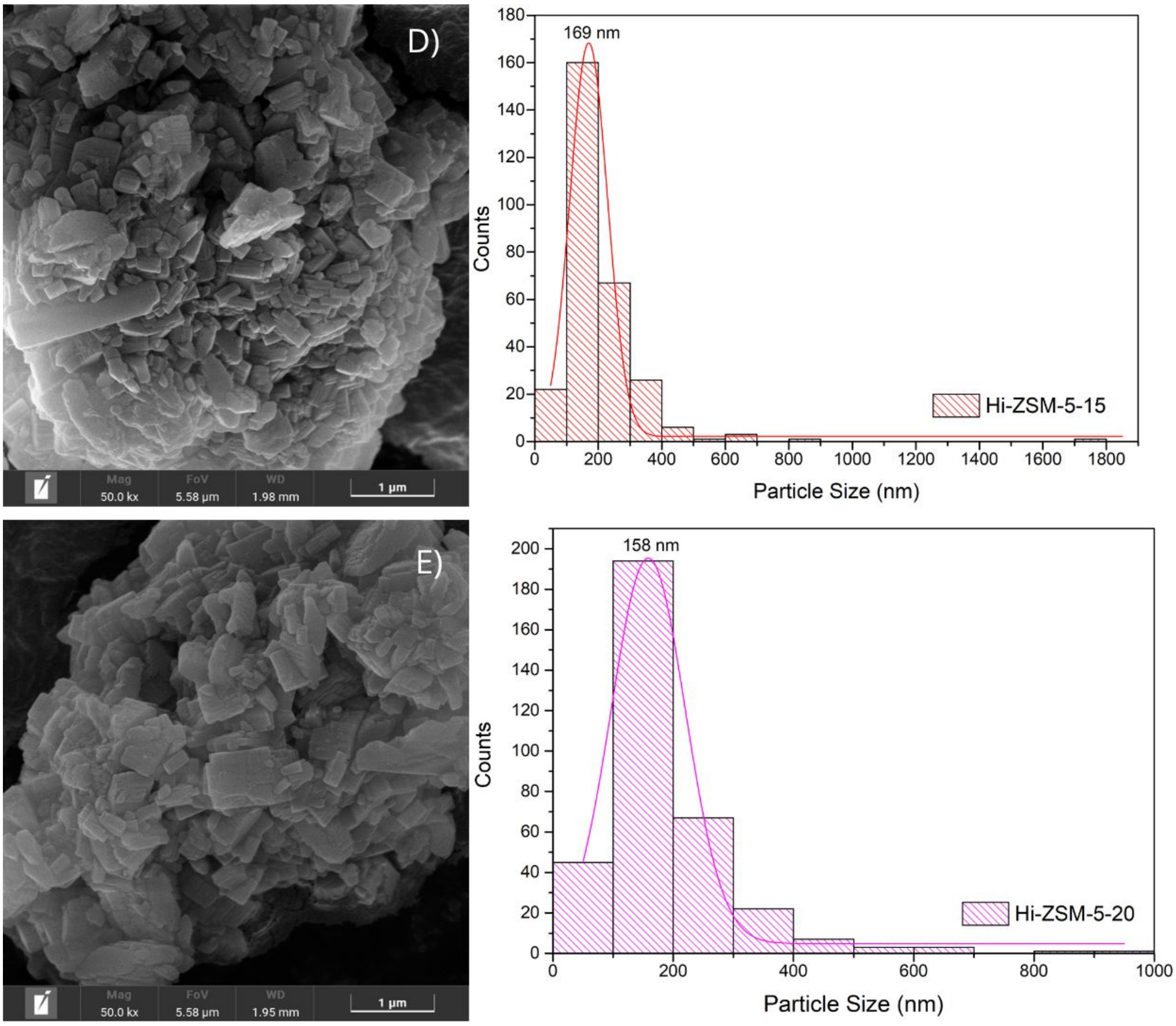

Figure S6. displays the SEM images of samples selected at different milling times under optimal conditions, along with their particle size distribution (PSD). Figure S6.A, which presents the commercial ZSM-5, shows crystals lacking a homogenous or well-defined structure, exhibiting irregular cuboid shapes and some particles with extended planar surfaces. These primary particles range widely from 100 nm up to 1500 nm, resulting in a high size dispersion and a mean particle size of 295 nm [1].

The initial 5 minutes of mechanical activation (Fig. S7.B), Hi-ZSM-5-5) were sufficient to induce the separation and breakage of the larger particles, consequently increasing the amount of smaller aggregates. This effect led to a measurable reduction in the overall average particle size to 205 nm and diminished the size dispersion (100 nm to 800 nm). These initial morphological changes are consistent with the XRD data and the proposed mechanochemical mechanism for initiating hierarchical zeolite formation.

Further increasing the milling time (Figs. S7. C, D, and E) continued to reduce the particle's average size, reaching a minimum of 158 nm (Hi-ZSM-5-20), with a distribution

ranging from 60 nm up to 1000 nm. However, prolonged milling also caused notable defects in the surface morphology; the number of nanoparticles increased alongside the formation of poorly defined shapes, such as flakes or slabs, generated by the flattening of nanoparticles during high-energy collisions [2]. The particle size data directly supports the XRD discussion on dynamic equilibrium: the average size increased slightly at 15 minutes (169 nm) before dropping again to 158 nm at 20 minutes. This variance, reflected in the fluctuations of relative crystallinity and crystallite size, confirms a clear process of destruction followed by recrystallization of the zeolite material.

Fig. S7. Thermal behavior of mechanochemical activation of zeolite ZSM-5 with co-templates (CTMA+ and TPA+) varying the time in the optimized condition of the ULTRA-TURRAX UTTD mechanochemical reactor.

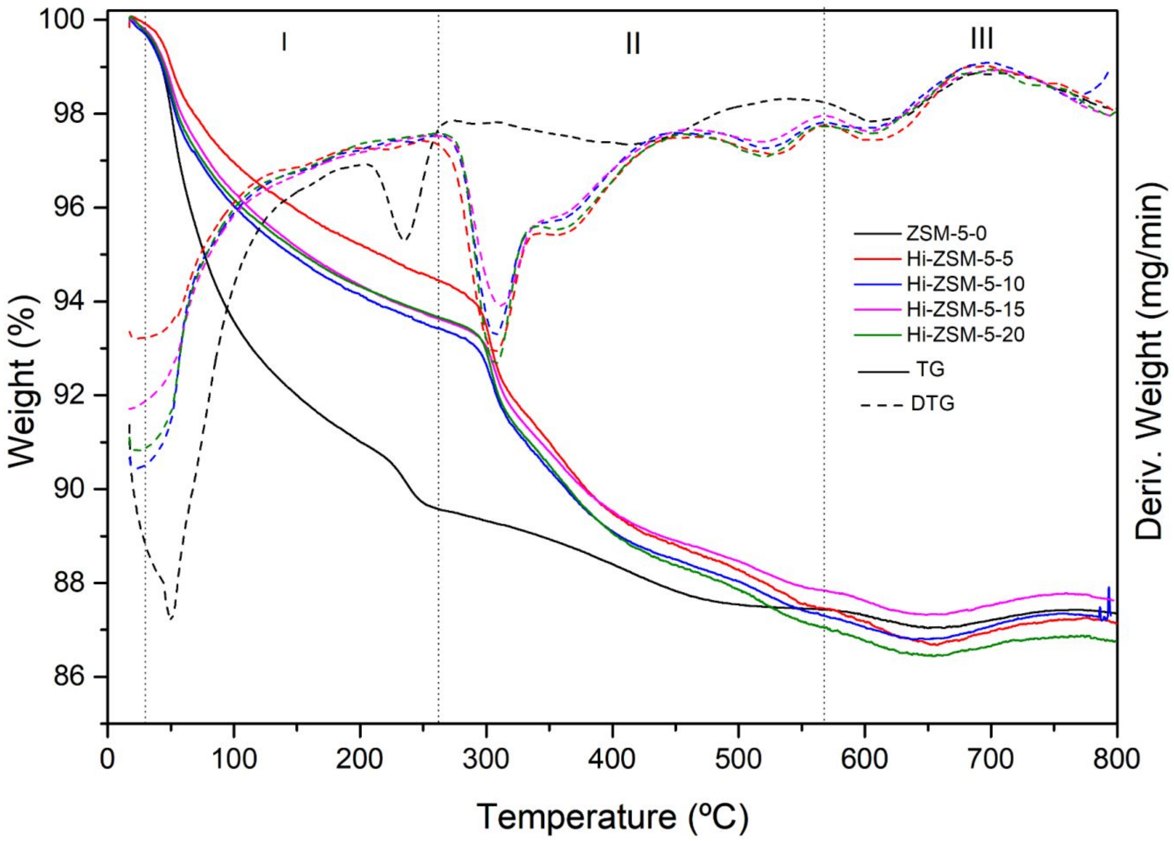

Figure S7. displays the TG and DTG plots for the ZSM-5 zeolite modified by mechanochemical co-template activation. For the commercial ammoniacal ZSM-5-0 zeolite, a total mass loss of approximately 10% is observed between 30 ºC and 260 ºC. The first maximum, observed at 50 ºC, is associated with the release of hygroscopic water adsorbed on the catalyst's external surface. A second endothermic peak occurs between 200 ºC and 260 ºC, reaching a maximum at 235 ºC. This event, associated with the desorption of physisorbed water located inside the ZSM-5 pores, accounts for an approximate 2% loss of the total mass. Between 450 ºC and 800 ºC, endothermic and exothermic events are observed; these are related to N_2 adsorption/desorption within the zeolite pores and are common to all analyzed samples, as seen in the n-butylamine thermodesorption study. [3, 4].

All analyzed samples share a common water loss region between 35 ºC and 160 ºC from the catalyst's external surface. While an increased heating rate accelerates the surface water

desorption process, the second DTG peak (associated with pure physisorbed water in the pores) is absent in the hierarchical zeolites.

Table S2.: Temperature ranges and mass losses of the commercial catalyst, ZSM-5-0 without templates and of the catalysts with mechanochemical activation with co-templates.

**Samples**	**Temperature Range (ºC)**			**Weight loss (%)**		
Eventos	I	II	III	I	II	III
ZSM-5-0	30-260	260-550	550-800	10,1	2	0,09
Hi-ZSM-5-5	30-260	260-550	550-800	5,4	7	0,4
Hi-ZSM-5-10	30-260	260-550	550-800	6,2	6,0	0,12
Hi-ZSM-5-15	30-260	260-550	550-800	6,1	5,7	0,28
Hi-ZSM-5-20	30-260	260-550	550-800	6,0	6,5	0,41

The weight loss event occurring between 280 ºC and 350 ºC is not solely attributable to the desorption of physisorbed water. This suggests that the remaining water is removed concurrently with the oxidative decomposition of the $CTMA^+$ and TPA^+ templates. Since all samples were prepared using ZSM-5 with a Si/Al ratio of 23, this

dehydration occurs in overlapping stages, as previously reported. This peak is specifically attributed to TPA^+ cation balancing the charge of Si-O groups at connectivity defects, rather than occluded TPA^+. This distinction is crucial because TPA^+ possesses a moderate basicity and becomes fully ionized in physisorbed water [5-7].

Table S2. provides a concise summary of the main mass loss events for all samples. It is important to highlight that a continuous mass degradation associated with water removal is observed across all analyzed samples up to 650 ºC. This continuous loss correlates with the varying strength of H_2O physisorption: 40 ºC–160 ºC for surface water and 160 ºC–650 ºC for water strongly retained within the internal pores [8]. The significant mass loss presented in the temperature range of 260 ºC–550 ºC is definitively attributed to the decomposition of the CTMA and TPA templates in the mechanochemically activated samples.

Overall, a 10% to 15% loss of total mass is observed during this template degradation process (as quantified in Table S2.). Considering that templates adsorbed merely on the external surface would have been removed during the initial filtration steps, this substantial remaining mass loss strongly indicates that the reaction between the templates and the zeolite occurred effectively within the interconnection of the porosity, The high temperatures (up to 650 ºC) are essential not only to remove the residual organic matter but also to break the Si-C bonds formed in the internal pores. This cleavage process is crucial, as it results in the formation of interconnected mesopores, thus explaining the enhanced porosity observed in the material. The proposed mechanism involves the mechanical activation dissolving the Si-O-Si bonds and subsequent recrystallization creating new bonds inside the porous framework.

These results demonstrate that the silanol groups on the catalyst surface are leached by the TPA^+ activation, allowing them to react with $CTMA^+$ at the pore interconnections. This generates a Si-C bond, thereby attaching a hydrophobic moiety to the surface via a covalent Si-C connection. The presence of these hydrophobic portions partially hinders the full development of zeolite crystals, leading to the creation of a nanocrystalline zeolite; subsequent calcination then produces the desired intercrystalline mesopores [9, 10].

Supporting References

1. Lima, D. S. D.; Zapelini, I. W.; Silva, L. L.; Mintova, S.; Martins, L. Impacts of Ball-Milling on ZSM-5 Zeolite Properties and Its Catalytic Activity in the Two-Phase Glycerol Ketalization with Acetone. *Catal. Today* **2024**, *441* (114842), 114842.
2. Saepurahman; Hashaikeh, R. Insight into Ball Milling for Size Reduction and Nanoparticles Production of H-Y Zeolite. *Mater. Chem. Phys.* **2018**, *220*, 322–330.
3. Cheng, Y. Preparation and characterization of nanosized ZSM-5 zeolites in the absence of organic template. *Materials Letters*, v. 59, n. **2005**, *27*, 3427–3430

4. Singh, M.; Kamble, R.; Viswanadham, N. Effect of Crystal Size on Physico-Chemical Properties of ZSM-5. *Catal. Letters* **2008**, *120* (3–4), 288–293.
5. Frantz, T. S.; Ruiz, W. A.; da Rosa, C. A.; Mortola, V. B. Synthesis of ZSM-5 with High Sodium Content for CO2 Adsorption. *Microporous Mesoporous Mater.* **2016**, *222*, 209–217.
6. Franco, A. M. M.; García, E. R.; Medina, R. L.; Ramírez, A. A. C. Properties and applications of natural zeolites. *Braz. J. Dev.* **2024**, *10* (1), 1713–1799.
7. Fernandes, J. O.; Neves, T. M.; da Silva, E. D.; da Rosa, C. A.; Mortola, V. B. Influence of Reaction Parameters on Glycerol Dehydration over HZSM-5 Catalyst. *React. Kinet. Mech. Catal.* **2021**, *132* (1), 485–498.
8. Knowlton, G. D.; White, T. R.; McKague, H. L. Thermal Study of Types of Water Associated with Clinoptilolite. *Clays Clay Miner.* **1981**, *29* (5), 403–411.
9. Hong-Ji, L. I.; Xiao-De, Z.; Jian-Min, Z.; Jing, Y.; Fa-Liang, W.; Xiang-Yang, W. CTAB on synthesis and pore structure of hierarchical zeolite. *Wuji Cailiao Xuebao (J. Inorganic Mater.)* **2018**, *33* (6), 629.
10. Antochshuk, V.; Araujo, A. S.; Jaroniec, M. Functionalized MCM-41 and CeMCM-41 Materials Synthesized via Interfacial Reactions. *J. Phys. Chem.* **2000**, *104* (41), 9713–9719.
